# Association Between Childhood Consumption of Ultraprocessed Food and Adiposity Trajectories in the Avon Longitudinal Study of Parents and Children Birth Cohort

**DOI:** 10.1001/jamapediatrics.2021.1573

**Published:** 2021-06-14

**Authors:** Kiara Chang, Neha Khandpur, Daniela Neri, Mathilde Touvier, Inge Huybrechts, Christopher Millett, Eszter P. Vamos

**Affiliations:** 1 Public Health Policy Evaluation Unit, Imperial College London, London, United Kingdom; 2 Department of Nutrition, School of Public Health, University of São Paulo, São Paulo, Brazil; 3 Center for Epidemiological Research in Nutrition and Health, School of Public Health, University of São Paulo, São Paulo, Brazil; 4 Department of Nutrition, Harvard T. H. Chan School of Public Health, Boston, Massachusetts; 5 Paris 13 University, Institut National de la Santé et de la Recherche Médicale U1153, INRA, Conservatoire National des Arts et Métiers, Nutritional Epidemiology Research Team, Epidemiology and Statistics Research Center–University of Paris, Bobigny, France; 6 Nutrition and Metabolism Branch, International Agency for Research on Cancer, Lyon, France

## Abstract

**Importance:**

Reports of associations between higher consumption of ultraprocessed foods (UPF) and elevated risks of obesity, noncommunicable diseases, and mortality in adults are increasing. However, associations of UPF consumption with long-term adiposity trajectories have never been investigated in children.

**Objective:**

To assess longitudinal associations between UPF consumption and adiposity trajectories from childhood to early adulthood.

**Design, Setting, and Participants:**

This prospective birth cohort study included children who participated in the Avon Longitudinal Study of Parents and Children (ALSPAC) in Avon County, southwest England. Children were followed up from 7 to 24 years of age during the study period from September 1, 1998, to October 31, 2017. Data were analyzed from March 1, 2020, to January 31, 2021.

**Exposures:**

Baseline dietary intake data were collected using 3-day food diaries. Consumption of UPF (applying the NOVA food classification system) was computed as a percentage of weight contribution in the total daily food intake for each participant and categorized into quintiles.

**Main Outcomes and Measures:**

Repeated recordings of objectively assessed anthropometrics (body mass index [BMI; calculated as weight in kilograms divided by height in meters squared], weight, and waist circumference) and dual-energy x-ray absorptiometry measurements (fat and lean mass indexes [calculated as fat and lean mass, respectively, divided by height in meters squared] and body fat percentage). Associations were evaluated using linear growth curve models and were adjusted for study covariates.

**Results:**

A total of 9025 children (4481 [49.7%] female and 4544 [50.3%] male) were followed up for a median of 10.2 (interquartile range, 5.2-16.4) years. The mean (SD) UPF consumption at baseline was 23.2% (5.0%) in quintile 1, 34.7% (2.5%) in quintile 2, 43.4% (2.5%) in quintile 3, 52.7% (2.8%) in quintile 4, and 67.8% (8.1%) in quintile 5. Among those in the highest quintile of UPF consumption compared with their lowest quintile counterpart, trajectories of BMI increased by an additional 0.06 (95% CI, 0.04-0.08) per year; fat mass index, by an additional 0.03 (95% CI, 0.01-0.05) per year; weight, by an additional 0.20 (95% CI, 0.11-0.28) kg per year; and waist circumference, by an additional 0.17 (95% CI, 0.11-0.22) cm per year.

**Conclusions and Relevance:**

These findings suggest that higher UPF consumption is associated with greater increases in adiposity from childhood to early adulthood. Robust public health measures that promote minimally processed foods and discourage UPF consumption among children are urgently needed to reduce obesity in England and globally.

## Key Points

**Question:**

Is consumption of ultraprocessed foods (UPF) in childhood associated with worse adiposity trajectories tracing into early adulthood?

**Findings:**

In this cohort study of 9025 British children, growth trajectories of body mass index, fat mass index, weight, and waist circumference from 7 to 24 years of age were greater among children with the highest (vs lowest) quintile of UPF consumption.

**Meaning:**

These findings suggest that radical and effective public health actions that reduce children’s exposure to and consumption of UPF and remove barriers to accessing minimally processed foods are urgently needed to counteract the growing burden of obesity in England and globally.

## Teaser

This cohort study assesses longitudinal associations between consumption of ultraprocessed food and adiposity trajectories from childhood to early adulthood among participants in the Avon Longitudinal Study of Parents and Children.

## Introduction

Growing evidence on the potentially harmful effects of ultraprocessed food (UPF) consumption on health has directed attention toward the public health significance of industrial food processing.^1,2,3,4,5,6,7,8^ Ultraprocessed foods, as defined by the NOVA food classification system, are industrial formulations of ingredients that undergo a series of physical, chemical, and biological processes.^9^ They typically lack intact healthy food components and include various additives.^9^ Ultraprocessed foods tend to be more energy-dense and nutritionally poorer (ie, high in levels of free sugar, salt, and saturated fats but low in levels of protein, dietary fiber, and micronutrients) compared with less processed alternatives and are designed to be cheap, palatable, durable, convenient, and appealing.^9^ These products are aggressively marketed by the food industry to promote purchasing and shape dietary preferences, and children are leading consumers of UPFs.^9,10^

The rapid expansion of global and industrialized food systems has gradually displaced traditional dietary patterns based on fresh and minimally processed foods, in favor of ready-to-eat UPFs.^9,10^ Currently, UPFs represent 65.4% and 66.2% of daily calorie intake among UK and US school-aged children, respectively.^11,12^ The growing consumption worldwide, including in low- and middle-income countries, has mirrored a parallel rise in the prevalence of childhood and adult obesity globally,^9,10,13^ suggesting that UPF consumption may be a key underlying driver of the obesity epidemic and diet-related noncommunicable diseases.^9,10,14,15^

A recent clinical trial found that UPF consumption leads to excess calorie intake and weight gain in adults,^1^ and cohort studies have reported associations between higher consumption and elevated risks of obesity,^2,3^ type 2 diabetes,^4,5^ cardiovascular disease,^6^ cancer,^7^ and mortality in adults.^8^ Associations of UPF consumption with adiposity in children and adolescents remain scarce, with only few previous small-scale studies available.^16,17,18,19,20^ This study investigates prospective associations between UPF consumption and objectively assessed adiposity measurements from childhood to early adulthood in a large cohort of British children.

## Methods

### Data Source

The Avon Longitudinal Study of Parents and Children (ALSPAC) is a prospective birth cohort study that initially enrolled 14 541 pregnant women residents in Avon, England, with an expected date of delivery between April 1, 1991, and December 31, 1992.^21,22^ Further enrollments after 1998 resulted in a sample of 14 888 children from singleton/twin pregnancies.^23^ In this study, children were followed up from 7 to 24 years of age during the study period from September 1, 1998, to October 31, 2017. Data were analyzed from March 1, 2020, to January 31, 2021. ALSPAC participants provided written informed consent, and ethical approval for the study was obtained from the ALSPAC Ethics and Law Committee and the local research ethics committees. This study followed the Strengthening the Reporting of Observational Studies in Epidemiology (STROBE) reporting guideline. The ALSPAC study website contains details of all data available through a fully searchable data dictionary and variable search tool (http://www.bristol.ac.uk/alspac/researchers/our-data/). Since 2014, study data were collected and managed using REDCap electronic data capture tools hosted at the University of Bristol, Bristol, UK.^24,25^

### Outcome Measures

Children were invited to a total of 10 clinic assessments almost annually from 7 to 17 years of age and then at 24 years of age (eTable 1 in the Supplement). Adiposity outcomes were measured following standardized procedures.^26^ Primary outcomes included body mass index (BMI), fat mass index (FMI), lean mass index (LMI), and percentage of total body fat. Secondary outcomes were BMI *z* score, weight, waist circumference, fat mass, and lean mass. Height was measured using a commercially available stadiometer (Harpenden; Holtain); weight, using a body fat analyzer (Tanita); and waist circumference, using a tape at the minimum circumference of the abdomen between iliac crests and lowest ribs.^26^ Total body fat and lean mass were assessed using a dual-energy x-ray absorptiometry scanner (Lunar Prodigy; GE Medical Systems).^26^ We computed BMI as weight in kilograms divided by height in meters squared. The FMI and LMI were calculated using dual-energy x-ray absorptiometry–measured fat mass and lean mass, respectively, divided by height in meters squared. Total body fat was computed as the percentage of fat mass divided by body mass. Age- and sex-standardized BMI *z* score was calculated for 7 to 17 years of age because the British 1990 Growth Reference is only available to 23 years of age.^27^ Completeness of adiposity outcomes ranged 89.5% to 99.9% in the study cohort. The mean number of repeated measurements was 6.5 for BMI, BMI *z* score, and weight; 5.3 for waist circumference; and 3.9 for FMI, LMI, fat mass, and lean mass.

### Dietary Exposure and Degree of Industrial Food Processing

A 3-day food diary was sent to parents before the child’s clinic assessment for parent completion at 7 years of age and child completion at 10 and 13 years of age.^26^ Respondents were instructed to record all food and beverage items the child consumed for 2 weekdays and 1 weekend day (not necessarily consecutive).^26^ Dietary data were reviewed by a nutritionist, and intakes were coded using the DIDO (Diet In, Data Out) computer program and linked to the McCance and Widdowson British food composition tables.^26,28^

We applied the NOVA food classification and categorized each food and beverage item into 1 of the 4 food groups based on their extent and purpose of industrial food processing^9^: (1) unprocessed/minimally processed foods are fresh, frozen, ground, pasteurized, or (nonalcoholic) fermented after separation from nature (eg, fruit, vegetable, milk, meat, legumes); (2) processed culinary ingredients are substances extracted from foods and used in common culinary preparation, cooking, and seasoning of group 1 foods (eg, table salt, sugar, vegetable oils, and butter); (3) processed foods are made by adding salt, sugar, or other group 2 ingredients to group 1 foods (eg, canned vegetables in brine, canned fish, freshly made breads and cheeses); and (4) UPFs are food and drink formulations of multiple substances, mostly of exclusive industrial use (eg, high-fructose corn syrup), and are manufactured through a series of complex industrial processes (eg, hydrogenation) and often contain cosmetic food additives (eg, colors, flavors, emulsifiers) that disguise any undesirable sensorial properties of the final product.^9^ Some examples are carbonated or dairy-based drinks, industrial-processed packaged breads with added preservatives or emulsifiers, and preprepared frozen or shelf-stable meals made with modified starches, stabilizers, or flavor enhancers (a full list of UPFs is presented in eFigure 1 in the Supplement).

### Study Covariates

Covariates included children’s age at clinic assessment, sex (male or female), race (White or non-White), birth weight (<2500, 2500-3999, or ≥4000 g), baseline physical activity (moderate to vigorous physical activity per day ≥60 minutes or otherwise), mean daily calorie intake (continuous), and quintiles of the Index of Multiple Deprivation 2004. The Index of Multiple Deprivation is the most common measure of deprivation for each small area of England based on 7 domains.^29^ Physical activity was based on the earliest recording of accelerometry data (collected at ages 11, 13, and 15 years) where children were instructed to wear a uniaxial accelerometer (model 7164; Actigraph) for 7 days. We categorized accelerometry data into 2 groups according to the UK government’s recommendation for children to accumulate at least 60 minutes of moderate to vigorous physical activity per day.^26,30,31^ Mothers’ self-reported data at baseline included prepregnancy BMI (<18.5, 18.5-24.9, 25.0-29.9, and ≥30.0), marital status (single or married/living with partner), highest educational attainment (Certificate of Secondary Education or none, vocational, O level, A level, or degree or above), and socioeconomic position based on the UK National Statistics Socioeconomic Classification (higher managerial, administrative, or professional; intermediate; or routine or manual occupation).^32^

### Statistical Analysis

Data were analyzed from March 1, 2020, to January 31, 2021. A total of 9025 children were included in the study after excluding 4581 children who did not participate in any clinic assessment, 1271 children with no dietary data, and 11 children with no outcome measurement at or before their dietary data collection (eFigure 2 in the Supplement). Those included were more likely to be female, White, and from higher socioeconomic backgrounds (eTable 2 in the Supplement). Each individual’s age at completion of their first dietary data collection was considered as the baseline; thus 7264 (80.5%) were followed up from 7 years of age; 1519 (16.8%), from 10 years of age; and 242 (2.7%), from 13 years of age. Moreover, their dietary data were based on a 1-day food diary for 727 children (8.0%), a 2-day food diary for 1171 children (13.0%), and a 3-day food diary for 7127 children (79.0%). For each child, we calculated the proportion of UPFs consumed in the total daily food intake and expressed as a percentage. This was considered the primary exposure because it better captures UPFs with zero-calorie content, such as artificially sweetened beverages. However, we also derived for sensitivity analysis a secondary exposure defined as the percentage of calorie contribution from UPFs relative to the total daily energy intake. We categorized individuals’ baseline UPF consumption into quintiles based on the cutoff points derived from dietary data at 7 years of age because most children were followed up from 7 years of age. We further compared this with quintiles derived from dietary data at 10 and 13 years of age. The quintiles were similar, and no sex-specific differences were identified. Time-varying exposure was not considered because even though a total of 7072 children (78.4%) provided follow-up dietary data, an absolute change in UPF consumption of 20% or greater was observed in only 1288 children (14.2%) between 7 and 10 years of age and 1831 children (20.2%) between 10 and 13 years of age.

Differences in baseline characteristics by UPF quintiles were compared using χ^2^ tests and analysis of variance where appropriate. Linear growth curve models were used to investigate the longitudinal associations between baseline UPF quintile and trajectories of adiposity outcomes. These 2-level linear regression models allow for individual-specific random intercept and random slope modeled with age as the underlying timescale. The models included 3 key variables: age, UPF quintile, and an interaction term between age and UPF quintile that examines the difference in mean growth trajectories of those in higher UPF quintiles compared with the lowest quintile reference group. We assessed nonlinearity by fitting a quadratic age term in both the fixed and random parts of the growth models. These terms were retained if there was evidence of improved model fit.

We used multiple imputation by chained equation to impute missing covariate data (range, 1.8%-27.7%) under the assumption of missing at random. Five imputed data sets were generated where the analytical models were performed on each, and the results were combined using the Rubin rule.^33^ Analyses based on complete data were conducted for comparison. Study covariates were included in a stepwise manner. Model 1 was not adjusted for any covariates; model 2 was adjusted for the child’s sex, race, birth weight, level of physical activity, and Index of Multiple Deprivation quintile; model 3 was additionally adjusted for the mother’s prepregnancy BMI, marital status, highest educational attainment, and socioeconomic position; and model 4 was additionally adjusted for the child’s baseline daily energy intake.

### Sensitivity Analyses

We performed a series of sensitivity analyses, including further adjustment for baseline fruit and vegetable intake; intakes of saturated fat, sugar, fiber, and sodium; restricting analyses to individuals with follow-up data; excluding twin children from the study cohort; stratifying by boys and girls; and recategorizing baseline UPF consumption into 5 groups per 20% absolute increment in their percentage of weight contribution toward daily food intake. All statistical analyses were performed using Stata SE, version 12.1 (StataCorp LLC). All statistical tests were 2 sided, and *P* < .05 was considered significant.

## Results

A total of 9025 children (4481 [49.7%] female and 4544 [50.3%] male) were followed up for a median of 10.2 (interquartile range, 5.2-16.4) years. The mean (SD) UPF consumption at baseline by quintile (Q1-Q5) was 23.2% (5.0%) of the total daily food intake in Q1 (lowest), 34.7% (2.5%) in Q2, 43.4% (2.5%) in Q3, 52.7% (2.8%) in Q4, and 67.8% (8.1%) in Q5 (highest) (eFigure 3 in the Supplement). Children assigned to differing UPF quintiles were not significantly different by sex, race, or birth weight (Table 1). However, children with higher UPF consumption were more likely to have lower maternal socioeconomic profiles compared with those in lower UPF quintiles (eg, 600 of 1858 [32.3%] for routine or manual occupation in Q5 vs 418 of 1708 [24.5%] in Q1). Major sources of UPFs among children in Q5 included fruit-based beverages (22.2%), carbonated beverages (11.5%), ready-to-eat/heat foods (8.6%), and industrial-processed breads and buns (5.9%) (eFigure 1 in the Supplement). By contrast, diets among children in Q1 were largely based on minimally processed foods, including water and tea (22.2%), milk and plain yogurt (20.2%), and fruit (6.0%).

**Table 1.** Sociodemographic Characteristics by Baseline Quintile of UPF Consumption Among 9025 ALSPAC Children (1998-2017), England

Characteristic	Study cohort^a^					
	Overall (N = 9025)	Quintile of baseline UPF consumption^b^				
		1 (n = 1708)	2 (n = 1759)	3 (n = 1923)	4 (n = 1777)	5 (n = 1858)
UPF consumption, mean (SD) [range], %	44.7 (15.9) [0-100]	23.2 (5.0) [0-29.9]	34.7 (2.5) [30.0-38.9]	43.4 (2.5) [39.0-47.9]	52.7 (2.8) [48.0-57.9]	67.8 (8.1) [58.0-100]
Total energy intake at baseline, mean (SD), kcal/d	1729 (347)	1698 (342)	1753 (345)	1737 (332)	1731 (335)	1726 (376)
Age at baseline, y^c^						
7	7264 (80.5)	1327 (77.7)	1435 (81.6)	1584 (82.4)	1460 (82.2)	1458 (78.5)
10	1519 (16.8)	292 (17.1)	270 (15.3)	296 (15.4)	297 (16.7)	364 (19.6)
13	242 (2.7)	89 (5.2)	54 (3.1)	43 (2.2)	20 (1.1)	36 (1.9)
Sex						
Male	4544 (50.3)	821 (48.1)	884 (50.3)	966 (50.2)	927 (52.2)	946 (50.9)
Female	4481 (49.7)	887 (51.9)	875 (49.7)	957 (49.8)	850 (47.8)	912 (49.1)
Race						
Non-White	780 (8.6)	152 (8.9)	165 (9.4)	170 (8.8)	157 (8.8)	136 (7.3)
White	8029 (90.0)	1512 (88.5)	1553 (88.3)	1704 (88.6)	1585 (89.2)	1675 (90.2)
Missing	216 (2.4)	44 (2.6)	41 (2.3)	49 (2.5)	35 (2.0)	47 (2.5)
Birth weight, g						
<2500	409 (4.5)	67 (3.9)	86 (4.9)	88 (4.6)	84 (4.7)	84 (4.5)
2500-3999	6905 (76.5)	1339 (78.4)	1337 (76.0)	1450 (75.4)	1385 (77.9)	1394 (75.0)
≥4000	1112 (12.3)	201 (11.8)	219 (12.5)	235 (12.2)	207 (11.6)	250 (13.5)
Missing	599 (6.6)	101 (5.9)	117 (6.7)	150 (7.8)	101 (5.7)	130 (7.0)
MVPA, min						
<60	4076 (45.2)	821 (48.1)	812 (46.2)	840 (43.7)	784 (44.1)	819 (44.1)
≥60	2453 (27.2)	468 (27.4)	476 (27.1)	542 (28.2)	481 (27.1)	486 (26.2)
Missing	2496 (27.7)	419 (24.5)	471 (26.8)	541 (28.1)	512 (28.8)	553 (29.8)
Index of multiple deprivation 2004, quintile						
1 (Least deprived)	2855 (31.6)	537 (31.4)	585 (33.3)	629 (32.8)	552 (31.1)	552 (29.7)
2	2113 (23.4)	460 (26.9)	413 (23.5)	454 (23.6)	404 (22.7)	382 (20.6)
3	1795 (19.9)	339 (19.8)	352 (20.0)	401 (20.9)	348 (19.6)	355 (19.1)
4	1198 (13.3)	192 (11.2)	217 (12.3)	222 (11.5)	267 (15.0)	300 (16.1)
5 (Most deprived)	899 (10.0)	142 (8.3)	161 (9.2)	180 (9.4)	177 (10.0)	239 (12.9)
Missing	165 (1.8)	38 (2.2)	31 (1.8)	37 (1.9)	29 (1.6)	30 (1.6)
Mother’s self-reported prepregnancy BMI						
Underweight (<18.5)	334 (3.7)	74 (4.3)	65 (3.7)	68 (3.5)	54 (3.0)	73 (3.9)
Normal (18.5-24.9)	5752 (63.7)	1153 (67.5)	1171 (66.6)	1203 (62.6)	1159 (65.2)	1066 (57.4)
Overweight (25.0-29.9)	1150 (12.7)	177 (10.4)	200 (11.4)	255 (13.3)	223 (12.5)	295 (15.9)
Obese (≥30.0)	393 (4.4)	48 (2.8)	63 (3.6)	88 (4.6)	88 (5.0)	106 (5.7)
Missing	1396 (15.5)	256 (15.0)	260 (14.8)	309 (16.1)	253 (14.2)	318 (17.1)
Mother’s marital status						
Single	1625 (18.0)	298 (17.4)	298 (16.9)	313 (16.3)	353 (19.9)	363 (19.5)
Married/living with partner	7203 (79.8)	1374 (80.4)	1423 (80.9)	1561 (81.2)	1393 (78.4)	1452 (78.1)
Missing	197 (2.2)	36 (2.1)	38 (2.2)	49 (2.5)	31 (1.7)	43 (2.3)
Mother’s highest educational attainment						
CSE/none	738 (8.2)	99 (5.8)	110 (6.3)	167 (8.7)	148 (8.3)	214 (11.5)
Vocational	662 (7.3)	92 (5.4)	123 (7.0)	119 (6.2)	144 (8.1)	184 (9.9)
O level	3189 (35.3)	468 (27.4)	560 (31.8)	700 (36.4)	696 (39.2)	765 (41.2)
A level	2421 (26.8)	497 (29.1)	529 (30.1)	497 (25.8)	470 (26.4)	428 (23.0)
Degree	1569 (17.4)	462 (27.0)	362 (20.6)	340 (17.7)	236 (13.3)	169 (9.1)
Missing	446 (4.9)	90 (5.3)	75 (4.3)	100 (5.2)	83 (4.7)	98 (5.3)
Mother’s NSSEC						
Higher managerial, administrative, and professional	2822 (31.3)	667 (39.1)	624 (35.5)	607 (31.6)	487 (27.4)	437 (23.5)
Intermediate occupations	2716 (30.1)	446 (26.1)	503 (28.6)	564 (29.3)	580 (32.6)	623 (33.5)
Routine and manual occupations	2598 (28.8)	418 (24.5)	479 (27.2)	557 (29.0)	544 (30.6)	600 (32.3)
Missing	889 (9.9)	177 (10.4)	153 (8.7)	195 (10.1)	166 (9.3)	198 (10.7)

Abbreviations: ALSPAC, Avon Longitudinal Study of Parents and Children; BMI, body mass index (calculated as weight in kilograms divided by height in square meters); CSE, Certificate of Secondary Education; MVPA, moderate to vigorous physical activity; NSSEC, National Statistics Socioeconomic Classification; UPF, ultraprocessed food.

^a^ Unless otherwise indicated, data are expressed as No. (%) of children. Percentages are rounded and may not total 100.

^b^ Quintile of UPF consumption was first computed for dietary data at 7, 10, and 13 years of age separately and was similar across waves; thus, a set of cutoff points for the baseline quintiles of UPF consumption was derived based on data from 7 years of age and defined at 30%, 39%, 48%, and 58% of daily food intake. Quintiles 1 and 5 indicate the lowest and highest UPF consumption, respectively.

^c^ Age when baseline UPF consumption was collected; >80% of children were followed up from 7 years of age.

**Figure 1.**
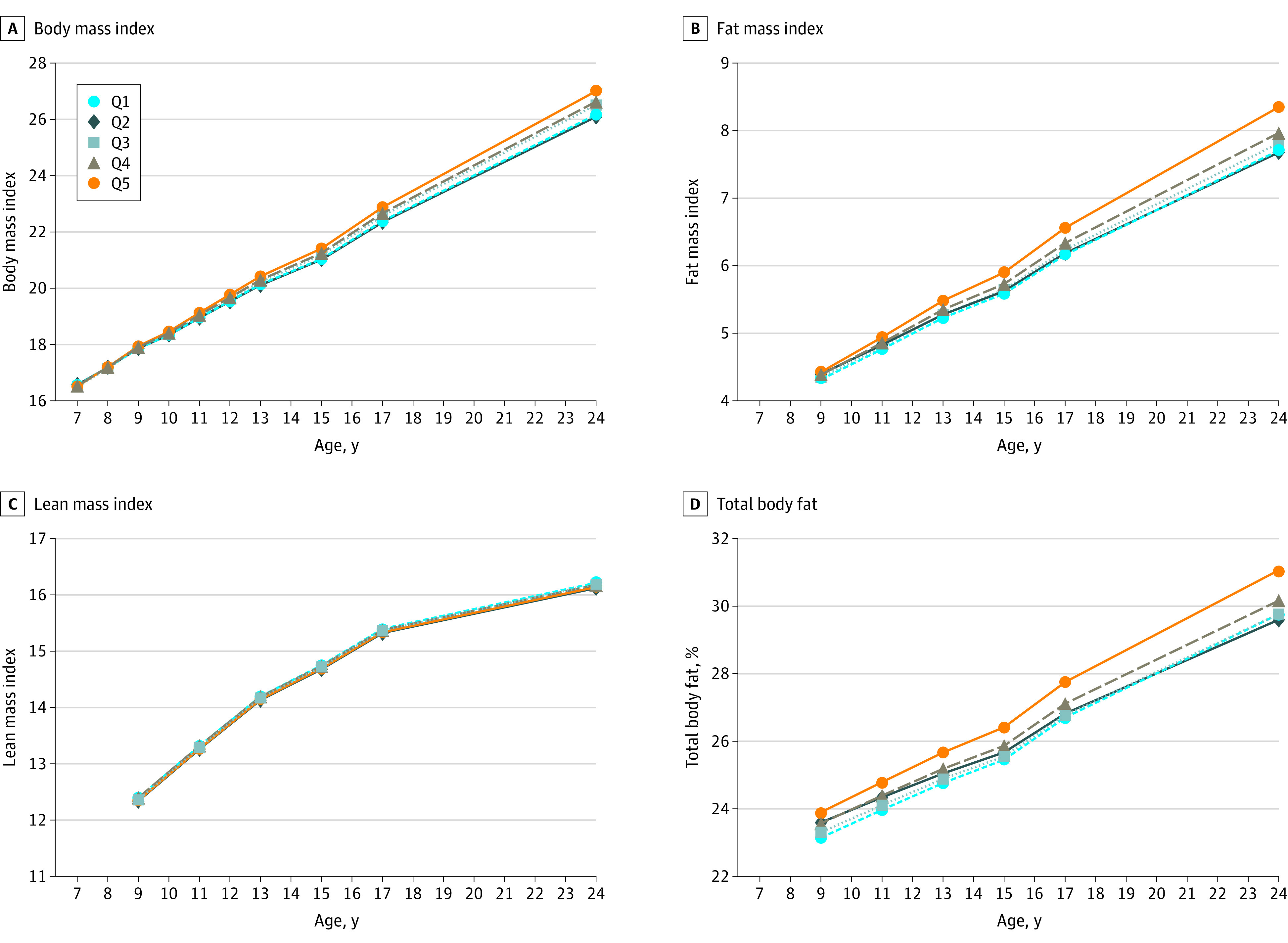
Trajectories of Primary Outcomes by Baseline Quintile of Ultraprocessed Food (UPF) Consumption Data are from 9025 children who participated in the Avon Longitudinal Study of Parents and Children. Percentage of daily food intake contributed by UPFs at baseline was categorized into quintiles (Q1-Q5, lowest to highest quintile of UPF consumption). Trajectories were plotted for the values estimated from the growth curve models at each age (wave) of clinical assessment. All linear growth models were fitted with individualized random intercept and random slope using age (and quadratic age for lean mass index outcome) as the underlying timescale and included baseline UPF quintile and an interaction term between age and baseline UPF quintile. Models were further adjusted for the child’s sex (male or female), race (White or non-White), birth weight (<2500, 2500-3999, or ≥4000 g), physical activity (moderate to vigorous physical activity per day ≥60 minutes or otherwise), quintiles of Index of Multiple Deprivation; the mother’s prepregnancy body mass index (BMI; calculated as weight in kilograms divided by height in square meters) (<18.5, 18.5-24.9, 25.0-29.9, or ≥30.0), marital status (single or married/living with partner), highest educational attainment (Certificate of Secondary Education or none, vocational, O level, A level, or degree or above), socioeconomic status based on UK National Statistics Socioeconomic Classification (higher managerial, administrative, and professional; intermediate; or routine and manual occupation); and the child’s total energy intake (continuous) at baseline. Baseline refers to 7 years of age for BMI and 9 years of age for fat or lean mass index (calculated as fat and lean mass, respectively, divided by height in meters squared), and percentage of body fat percentage outcomes.

Findings from the growth models remained consistent while adjusting for covariates in multiple steps (eTables 3 and 4 in the Supplement). Fully adjusted results for the longitudinal associations between baseline UPF quintile and adiposity outcomes are presented in Table 2, and the fitted trajectories of primary adiposity outcomes are shown in Figure 1. Mean BMI at baseline (7 years of age) did not significantly differ across baseline UPF quintiles (eg, β, 0.08 [95% CI, −0.09 to 0.24] for Q5 vs Q1). Mean BMI among children in Q1 increased by 0.55 (95% CI, 0.53-0.56) per year. However, increases in BMI were significantly greater among the 3 highest UPF quintiles with a dose-response association (eg, BMI increased by an additional 0.06 [95% CI, 0.04-0.08] per year in Q5 compared with Q1).

**Table 2.** Longitudinal Associations Between Baseline UPF Consumption and Adiposity Among 9025 ALSPAC Children (1998-2017), England

Term	β coefficient (95% CI)^a^								
	BMI (n = 9020)	FMI (n = 8078)	Total fat, % (n = 8085)	LMI (n = 8078)	Weight, kg (n = 9012)	Waist circumference, cm (n = 9021)	BMI *z* score (n = 9018)	Fat mass, kg (n = 8085)	Lean mass, kg (n = 8085)
Baseline UPF^b^									
Q1	0 [Reference]	0 [Reference]	0 [Reference]	0 [Reference]	0 [Reference]	0 [Reference]	0 [Reference]	0 [Reference]	0 [Reference]
Q2	0.06 (−0.10 to 0.23)	0.08 (−0.09 to 0.26)	0.65 (−0.01 to 1.30)	0.005 (−0.06 to 0.07)	0.35 (0.007 to 0.69)^c^	0.26 (−0.14 to 0.66)	0.06 (−0.01 to 0.13)	0.11 (−0.31 to 0.52)	0.13 (−0.16 to 0.42)
Q3	0.006 (−0.16 to 0.17)	0.11 (−0.06 to 0.28)	0.67 (0.02 to 1.32)^c^	0.009 (−0.06 to 0.07)	0.30 (−0.03 to 0.63)	0.03 (−0.36 to 0.42)	0.03 (−0.04 to 0.10)	0.10 (−0.32 to 0.51)	−0.01 (−0.30 to 0.28)
Q4	0.02 (−0.15 to 0.19)	0.17 (−0.01 to 0.34)	1.02 (0.35 to 1.67)^d^	−0.01 (−0.08 to 0.05)	0.34 (−0.007 to 0.68)	0.22 (−0.18 to 0.62)	0.05 (−0.02 to 0.12)	0.20 (−0.22 to 0.62)	−0.07 (−0.36 to 0.23)
Q5	0.08 (−0.09 to 0.24)	0.27 (0.09 to 0.45)^d^	1.47 (0.81 to 2.13)^d^	−0.01 (−0.08 to 0.05)	0.30 (−0.04 to 0.65)	0.16 (−0.25 to 0.56)	0.05 (−0.02 to 0.12)	0.51 (0.08 to 0.93)^c^	0.07 (−0.23 to 0.37)
Age, per year^e^	0.55 (0.53 to 0.56)^d^	0.22 (0.20 to 0.23)^d^	0.39 (0.35 to 0.43)^d^	0.55 (0.53 to 0.55)^d^	5.46 (5.38 to 5.53)^d^	3.36 (3.30 to 3.41)^d^	0.02 (0.01 to 0.02)^d^	0.96 (0.92 to 1.00)^d^	4.44 (4.38 to 4.49)^d^
Quadratic age per year^e^	NA	NA	NA	−0.02 (−0.02 to −0.01)^d^	−0.12 (−0.12 to −0.11)^d^	−0.11 (−0.11 to −0.10)^d^	NA	NA	−0.17 (−0.17 to −0.16)^d^
Interaction^f^									
Q1 × age	0 [Reference]	0 [Reference]	0 [Reference]	0 [Reference]	0 [Reference]	0 [Reference]	0 [Reference]	0 [Reference]	0 [Reference]
Q2 × age	0.02 (−0.001 to 0.04)	0.005 (−0.01 to 0.02)	−0.03 (−0.08 to 0.02)	0.008 (−0.003 to 0.01)	0.06 (−0.02 to 0.14)	0.05 (−0.008 to 0.10)	0.0003 (−0.006 to 0.007)	0.03 (−0.02 to 0.09)	0.02 (−0.04 to 0.08)
Q3 × age	0.03 (0.005 to 0.04)^c^	0.01 (−0.01 to 0.02)	−0.02 (−0.07 to 0.03)	−0.003 (−0.01 to 0.008)	0.04 (−0.03 to 0.12)	0.06 (0.006 to 0.11)^c^	0.002 (−0.005 to 0.009)	0.06 (−0.003 to 0.11)	−0.007 (−0.07 to 0.05)
Q4 × age	0.04 (0.01 to 0.06)^d^	0.01 (−0.01 to 0.03)	−0.04 (−0.10 to 0.01)	0.009 (−0.002 to 0.02)	0.10 (0.01 to 0.18)^c^	0.08 (0.02 to 0.14)^d^	0.003 (−0.004 to 0.009)	0.07 (0.01 to 0.13)^c^	0.03 (−0.03 to 0.10)
Q5 × age	0.06 (0.04 to 0.08)^d^	0.03 (0.01 to 0.05)^d^	0.004 (−0.05 to 0.06)	0.004 (−0.007 to 0.01)	0.20 (0.11 to 0.28)^d^	0.17 (0.11 to 0.22)^d^	0.01 (0.003 to 0.01)^d^	0.15 (0.08 to 0.21)^d^	−0.04 (−0.11 to 0.02)

Abbreviations: ALSPAC, Avon Longitudinal Study of Parents and Children; BMI, body mass index (calculated as weight in kilograms divided by height in meters squared); FMI, fat mass index (calculated as fat mass divided by height in meters squared); LMI, lean mass index (calculated as lean mass divided by height in meters squared); NA, not applicable; UPF, ultraprocessed food.

^a^ Linear growth curve models were used with individualized random intercept and random slope using age (and quadratic age where appropriate) as the underlying timescale, included baseline UPF quintile and an interaction term between age and baseline UPF quintile, and were further adjusted for the child’s sex (male or female), race (White or non-White), birth weight (<2500, 2500-3999, or ≥4000 g), physical activity (moderate to vigorous physical activity per day ≥60 minutes or otherwise), quintiles of Index of Multiple Deprivation; the mother’s prepregnancy BMI (<18.5, 18.5-24.9, 25.0-29.9, or ≥30.0), marital status (single or married/living with partner), highest educational attainment (Certificate of Secondary Education or none, vocational, O level, A level or degree or above), socioeconomic status based on UK National Statistics Socioeconomic Classification (higher managerial, administrative, and professional; intermediate; or routine and manual occupation); and the child’s total energy intake (continuous) at baseline. Baseline refers to 7 years of age for BMI, BMI *z* score, weight, and waist circumference outcomes and 9 years of age for fat and lean mass indexes, fat and lean mass, and percentage of body fat outcomes. Ultraprocessed food consumption was defined as the proportion of its weight contribution relative to daily food intake and was categorized into quintiles, with the lowest quintile (Q1) representing the lowest UPF consumption and the highest quintile (Q5) representing the highest UPF consumption.

^b^ Coefficient of baseline UPF quintile assesses the difference in mean adiposity outcomes at baseline among those in the higher UPF consumption quintiles compared with the lowest UPF quintile reference group.

^c^ *P* < .05.

^d^ *P* < .01.

^e^ Coefficient of age and quadratic age captures the average yearly growth in adiposity outcomes for the reference group and were centered at baseline age of each outcome (described above).

^f^ Coefficient of interaction term examines the difference in mean growth trajectories among those in the higher UPF consumption quintiles compared with the lowest UPF quintile reference group.

The mean FMI at baseline (9 years of age) was significantly higher in Q5 by 0.27 (95% CI, 0.09-0.45) compared with Q1. The mean FMI increased by 0.22 (95% CI, 0.20-0.23) per year in Q1, and this growth trajectory was found significantly greater in Q5 than Q1 by an additional 0.03 (95% CI, 0.01-0.05) per year. Mean body fat percentage at baseline (9 years of age) was significantly higher among children of the 3 highest UPF quintiles (eg, 1.47% [95% CI, 0.81%-2.13%] higher in Q5 compared with Q1). However, the growing trajectories of body fat percentage were not significantly different across UPF quintiles. Mean LMI was estimated to grow at an annual rate of 0.55 – (2 × 0.02 × follow-up years) from 9 years of age, but neither the LMI at 9 years of age nor its growth trajectory was found significantly different among children of varying UPF quintiles.

**Figure 2.**
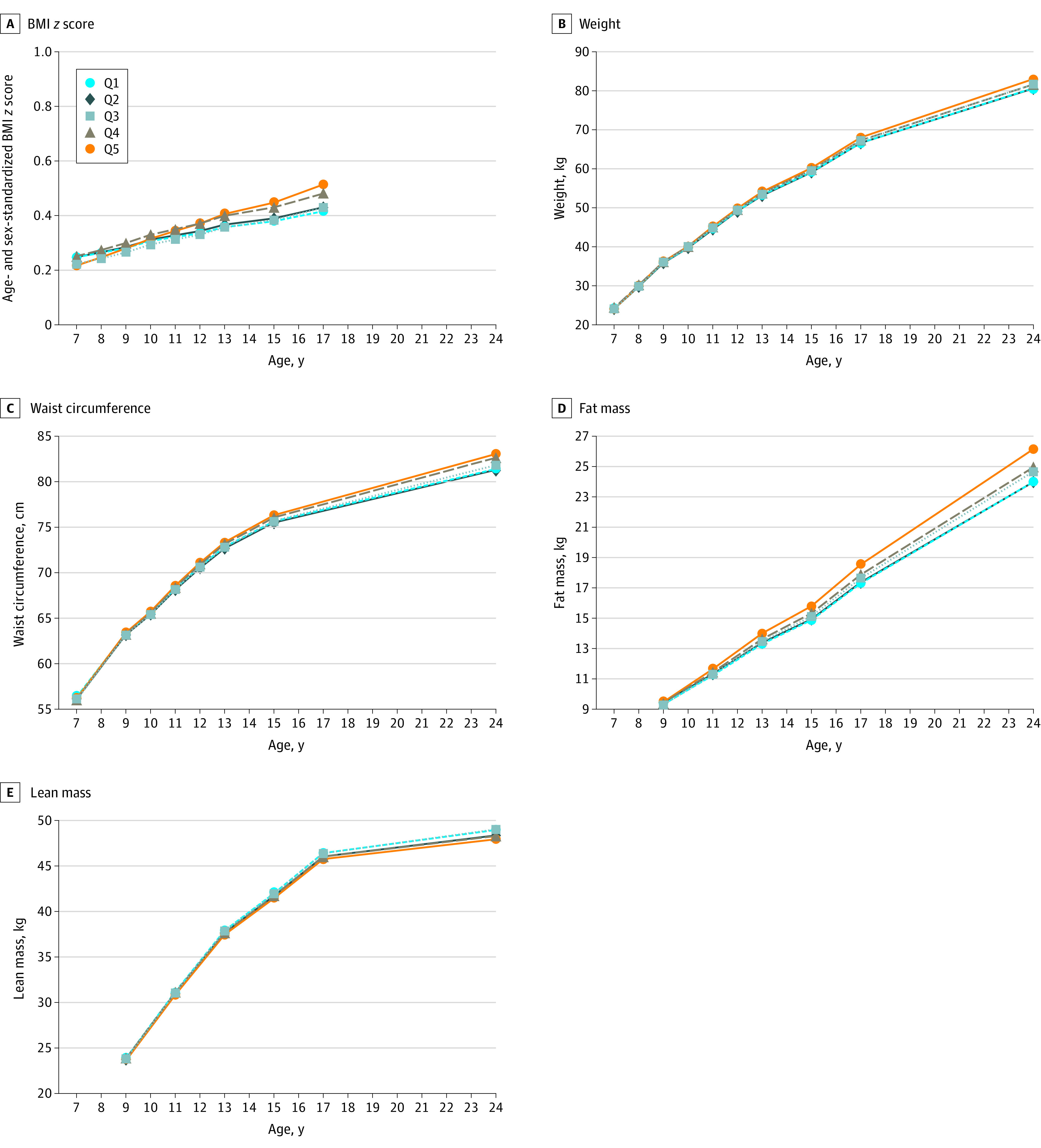
Trajectories of Secondary Outcomes by Baseline Quintile of Ultraprocessed Food (UPF) Consumption Data are from 9025 children who participated in the Avon Longitudinal Study of Parents and Children. Percentage of daily food intake contributed by UPFs at baseline was further categorized into quintiles (Q1-Q5, lowest to highest quintile of UPF consumption). Trajectories were plotted for the values estimated from the growth curve models at each age (wave) of clinic assessment. All linear growth models were fitted with individualized random intercept and random slope using age (and quadratic age for weight, waist circumference, and lean mass outcomes) as the underlying timescale and included baseline UPF quintile and an interaction term between age and baseline UPF quintile. Models were further adjusted for the child’s sex (male or female), race (White or non-White), birth weight (<2500, 2500-3999, or ≥4000 g), physical activity (moderate to vigorous physical activity per day ≥60 minutes or otherwise), quintiles of Index of Multiple Deprivation; the mother’s prepregnancy body mass index (BMI; calculated as weight in kilograms divided by height in square meters) (<18.5, 18.5-24.9, 25.0-29.9, or ≥30.0), marital status (single or married/living with partner), highest educational attainment (Certificate of Secondary Education or none, vocational, O level, A level, or degree or above), socioeconomic status based on UK National Statistics Socioeconomic Classification (higher managerial, administrative, and professional; intermediate; or routine and manual occupation); and the child’s total energy intake (continuous) at baseline. Baseline refers to BMI *z* score, weight, and waist circumference outcomes at 7 years of age and fat and lean mass and percentage of body fat outcomes at 9 years of age.

Mean levels of BMI *z* score, weight, and waist circumference were not significantly different at baseline (7 years of age) across UPF quintiles except for weight among children in Q2 (β = 0.35 [95% CI, 0.007-0.69]) (Table 2 and Figure 2). However, when compared with children in Q1, increases in weight and waist circumference trajectories were significantly greater in the 2 and 3 highest UPF quintiles, respectively, with a dose-response association (eg, mean weight increased by an additional 0.10 [95% CI, 0.01-0.18] kg per year in Q4 compared with Q1 and by an additional 0.20 [95% CI, 0.11-0.28] kg per year in Q5 compared with Q1). Trajectories of BMI *z* score were only significantly greater in Q5 (β = 0.01 [95% CI, 0.003-0.01]). Results for fat mass and lean mass were similar to FMI and LMI findings, respectively. By 24 years of age, significantly greater mean levels of BMI by 1.18 (95% CI, 0.78-1.57), FMI by 0.78 (95% CI, 0.46-1.08), body fat percentage by 1.53% (95% CI, 0.81%-2.25%), weight by 3.66 (95% CI, 2.18-5.12) kg, and waist circumference by 3.08 (95% CI, 2.08-4.06) cm were observed in Q5 compared with Q1.

Results of sensitivity analyses were largely consistent with the main findings (eTables 5 and 6 and eFigures 4-6 in the Supplement). Girls were observed with a steeper trajectory of body fat measures than boys, although their BMI trajectories were similar. Analyses using the secondary exposure showed that the mean UPF consumption in the study cohort was 61.4% of the daily energy intake, and major contributors of energy intake were ready-to-eat/heat UPF and industrial-processed breads and buns.

## Discussion

In this large prospective study following up British children from 7 to 24 years, growth trajectories among children with the highest (vs lowest) UPF consumption increased by an additional 0.06 (95% CI, 0.04-0.08) per year for BMI, 0.03 (95% CI, 0.01-0.05) per year for FMI, 0.20 (95% CI, 0.11-0.28) kg per year for weight, and 0.17 (95% CI, 0.11-0.22) cm per year for waist circumference. Dose-response associations were observed consistently for BMI, weight, and waist circumference trajectories among those in the 2 highest UPF quintiles. By 24 years of age, children with the highest (vs lowest) UPF consumption were observed to have greater BMI by 1.18 (95% CI, 0.78-1.57), greater FMI by 0.78 (95% CI, 0.46-1.08), and greater body fat percentage by 1.53% (95% CI, 0.81%-2.25%).

Previous cohort studies of children/adolescents (sample size, 307-3454 participants)^16,17,18,19,20^ had shorter follow-up and yielded inconsistent findings. Two studies^16,17^ found no significant associations between UPF consumption at 4 years of age and BMI measures 3 to 4 years later, whereas 1 study^20^ reported no differences in BMI growth from 16 to 18 years of age. However, a Portuguese study^19^ reported a 0.028 increase in BMI *z* score at 10 years of age per 100-kcal/d higher UPF consumption at 4 years of age, and a Brazilian study^18^ reported a 0.20 increase in BMI and 0.14 increase in FMI, from 6 to 11 years of age per 100-g/d increase in UPF consumption. Our findings were based on multiple adiposity measurements from 7 to 24 years of age and detailed 3-day food diaries, whereas previous studies were largely based on food frequency questionnaires that may have limited ability to accurately capture UPFs. Notably, British children have a high UPF consumption compared with previous studies based in Brazil,^16,18^ Portugal,^19^ or Spain^17^ (range, 27.3%-42.0% of daily calorie intake). The positive longitudinal association between childhood consumption of sugar-sweetened beverages and adiposity has been widely documented^34^; our results are reflective of this because sugar-sweetened and artificially sweetened beverages constituted a great proportion of UPF consumption, especially in those with the highest quintile of consumption (33.7%).

The increasing availability and variety of UPFs have reshaped global food systems by displacing dietary patterns previously based on fresh and minimally processed foods.^9,10^ Of particular concern is the growing consumption of UPFs among children and adolescents, who are leading consumers, including in middle-income countries.^11,12,35,36^ These findings have major public health implications, with higher UPF consumption associated with excess calorie intake^1^ and elevated risk of obesity,^2,3^ type 2 diabetes,^4,5^ hypertension,^37^ cardiovascular disease,^6^ cancer,^7^ and mortality.^8^ Our findings add positive associations between UPF consumption and adiposity outcomes throughout childhood, which is crucially important given that lifelong dietary patterns develop from childhood and may lead to widespread consequences on health and well-being throughout the life course.^38^

The UPF industry is highly profitable through the use of low-cost supply chains and aggressive marketing strategies to promote excess consumption.^14,15^ Global economic policies and trade agreements that favor the interests of transnational food corporations have further enhanced their central role in the global transformation of food systems and have undermined implementation of effective policies to curb UPF consumption.^10,15^ Nevertheless, policies are emerging that explicitly target UPFs.^10^ Public health authorities in Brazil, Uruguay, Ecuador, Peru, France, Canada, and Israel have amended their national dietary guidelines with recommendations to limit UPF consumption.^10,39,40^ France has set an ambitious target to reduce UPF consumption by 20% by 2022. Action on UPFs in the UK and elsewhere remains limited, instead emphasizing the reduction of certain nutrients.^14,41^ Voluntary product reformulations have been shown to be ineffective,^10,41^ and even bolder regulations may not address health harms, because they may overlook several UPFs (eg, artificially sweetened beverages) that contain industrial trans-fatty acids,^42^ food additives, or toxic contaminants,^43,44^ even when their calorie, salt, and sugar content are reduced. Only mandatory policies that target UPFs holistically, with globally cooperative strengthening of regulations and trade agreements to reduce the supply and consumption of UPFs, will counteract the substantial burden of UPF consumption on the environment and health care systems worldwide.^14,41,45^

### Limitations

Our study has several limitations. First, some individuals had fewer adiposity measurements collected, and no data collection was conducted between 17 and 24 years of age. However, completeness of outcome data was high in the study cohort (89.5%-99.9%), and a mean of 3.9 to 6.5 repeated measurements across study outcomes were available. Second, misclassification of food/beverage items by the NOVA classification may occur, but this is likely minimal given the detailed food diaries used. Third, major changes in UPF consumption may contribute to a shift in adiposity trajectories, but we did not use a time-varying exposure because of the modest changes in UPF consumption from 7 to 13 years of age. Fourth, availability of multiple food diaries lowers measurement bias, and only 727 (8.0%) of the cohort completed on a single occasion, whereas most participants completed 2 or more days. Fifth, we examined potential dietary misreporting based on the ratio of energy intake to estimated energy expenditure.^46^ The results remained closely consistent after the exclusion of 1314 underreporters (14.6%) and 715 overreporters (7.9%). Sixth, missing data may introduce bias, but we used multiple imputation, whereas auxiliary variables were included as appropriate. A comparison of main findings with those from complete case analyses yielded similar results. Finally, although we accounted for a wide range of factors, the observational nature of the study means that residual confounding may have affected our results.

## Conclusions

The findings of this cohort study suggest that higher consumption of UPFs in childhood is associated with more rapid progression of BMI, FMI, weight, and waist circumference into adolescence and early adulthood. More radical and effective public health actions that reduce children’s exposure and consumption of UPFs are urgently needed to address childhood obesity in England and internationally.

## Notes

Supplement. **eTable 1.** Timing of Outcome Data Collection Among 9025 ALSPAC Children (1998-2017), England **eTable 2.** Comparison of ALSPAC Participants Included and Excluded From This Study **eTable 3.** Stepwise Adjustments for Body Mass Index and Fat Mass Index **eTable 4.** Stepwise Adjustments for Lean Mass Index and Body Fat Percentage **eTable 5.** Results of Sensitivity Analyses for Primary Adiposity Outcomes **eTable 6.** Results of Growth-Curve Model Using Secondary Exposure **eFigure 1.** Sources of Ultraprocessed Foods and Minimally Processed Foods **eFigure 2.** Flowchart for Study Cohort Inclusion **eFigure 3.** Mean Percentage of Daily Food Intake Stratified by Quintiles of Ultraprocessed Food Consumption **eFigure 4.** Trajectories of Body Mass Index Stratified by Sex **eFigure 5.** Sources of Ultraprocessed Foods and Minimally Processed Foods Based on Dietary Energy Intake **eFigure 6.** Results of Growth-Curve Model Using Secondary Exposure
