# Supplementary material for: Association Between Childhood Consumption of Ultraprocessed Food and Adiposity Trajectories in the Avon Longitudinal Study of Parents and Children Birth Cohort
Source: JAMA Pediatr. 2021 Jun 14;175(9):e211573. doi: 10.1001/jamapediatrics.2021.1573 (PMC8424476; doi:10.1001/jamapediatrics.2021.1573)
Supplement: Supplement. — eTable 1. Timing of Outcome Data Collection Among 9025 ALSPAC Children (1998-2017), England eTable 2. Comparison of ALSPAC Participants Included and Excluded From This Study eTable 3. Stepwise Adjustments for Body Mass Index and Fat Mass Index eTable 4. Stepwise Adjustments for Lean Mass Index and Body Fat Percentage eTable 5. Results of Sensitivity Analyses for Primary Adiposity Outcomes eTable 6. Results of Growth-Curve Model Using Secondary Exposure eFigure 1. Sources of Ultraprocessed Foods and Minimally Processed Foods eFigure 2. Flowchart for Study Cohort Inclusion eFigure 3. Mean Percentage of Daily Food Intake Stratified by Quintiles of Ultraprocessed Food Consumption eFigure 4. Trajectories of Body Mass Index Stratified by Sex eFigure 5. Sources of Ultraprocessed Foods and Minimally Processed Foods Based on Dietary Energy Intake eFigure 6. Results of Growth-Curve Model Using Secondary Exposure [file jamapediatr-e211573-s001.pdf]

Supplementary Online Content

Chang K, Khandpur N, Neri D, et al. Association between childhood consumption of ultraprocessed food and adiposity trajectories in the Avon Longitudinal Study of Parents and Children birth cohort. *JAMA Pediatr*. Published online June 14, 2021.
doi:10.1001/jamapediatrics.2021.1573

eTable 1. Timing of Outcome Data Collection Among 9025 ALSPAC Children (1998-2017), England

eTable 2. Comparison of ALSPAC Participants Included and Excluded From This Study

eTable 3. Stepwise Adjustments for Body Mass Index and Fat Mass Index

eTable 4. Stepwise Adjustments for Lean Mass Index and Body Fat Percentage

eTable 5. Results of Sensitivity Analyses for Primary Adiposity Outcomes

eTable 6. Results of Growth-Curve Model Using Secondary Exposure

eFigure 1. Sources of Ultraprocessed Foods and Minimally Processed Foods

eFigure 2. Flowchart for Study Cohort Inclusion

eFigure 3. Mean Percentage of Daily Food Intake Stratified by Quintiles of Ultraprocessed Food Consumption

eFigure 4. Trajectories of Body Mass Index Stratified by Sex

eFigure 5. Sources of Ultraprocessed Foods and Minimally Processed Foods Based on Dietary Energy Intake

eFigure 6. Results of Growth-Curve Model Using Secondary Exposure

This supplementary material has been provided by the authors to give readers additional information about their work.

© 2021 Chang K et al. *JAMA Pediatrics*.

eTable 1. Timing of Outcome Data Collection Among 9025 ALSPAC Children (1998-2017), England

Clinic age	Height	Weight	Waist Circumference	DXA Fat/Fat-free mass
7	v	v	v	
8	v	v		
9	v	v	v	v
10	v	v	v	
11	v	v	v	v
12	v	v	v	
13	v	v	v	v
15	v	v	v	v
17	v	v		v
24	v	v	v	v

Abbreviation: DXA, dual-energy X-ray absorptiometry.

© 2021 Chang K et al. *JAMA Pediatrics*.

eTable 2. Comparison of ALSPAC Participants Included and Excluded From This Study

	Participants Included in analytical sample	Participants with no clinical data	*P* value[a]	Participants with no dietary data	*P* value[b]	Total participants excluded	*P* value[c]
	n=9025	*n=4592*		*n=1271*		*n=5863*	
**Sex (%)**							
Male	50.3%	54.6%	<.001	43.4%	<.001	52.1%	.001
Female	49.7%	45.3%		56.6%		47.7%	
Missing	0%	0.1%		0.1%		0.1%	
**Ethnicity (%)**							
Non-white	8.6%	4.6%	<.001	5.2%	<.001	2.7%	<.001
White	89.0%	68.7%		66.5%		68.2%	
Missing	2.4%	26.7%		28.3%		27.0%	
**Mother's highest educational attainment (%)**							
CSE/none	8.2%	21.4%	<.001	16.1%	<.001	20.2%	<.001
Vocational	7.3%	8.1%		7.8%		8.0%	
O level	35.3%	26.3%		25.7%		26.1%	
A level	26.8%	13.2%		13.5%		13.2%	
Degree	17.4%	6.4%		7.9%		6.7%	
Missing	4.9%	24.5%		29.1%		25.5%	
**Mother's NSSEC (%)**							
1. Higher managerial, administrative and professional	31.3%	16.6%	<.001	17.5%	<.001	16.7%	<.001
2. Intermediate	30.1%	21.6%		17.9%		20.7%	
3. Routine and manual	28.8%	36.6%		33.4%		35.9%	
Missing	9.9%	25.2%		31.2%		26.5%	

Abbreviations: NSSEC, (UK) National Statistics Socioeconomic Classification.

[a] Chi-square tests were used to compare socio-demographic characteristics between participants included and those excluded who had no clinical data collected between ages 7-24 years.

[b] Chi-square tests were used to compare socio-demographic characteristics between participants included and those excluded who had no food diary data submitted.

[c] Chi-square tests were used to compare socio-demographic characteristics between participants included and those excluded who had no clinical data or food diary data recorded.

© 2021 Chang K et al. *JAMA Pediatrics*.

eTable 3. Stepwise Adjustments for Body Mass Index and Fat Mass Index

**Body Mass Index (kg/m²)**		**Model 1[a,b]**	**Model 2[a,c]**	**Model 3[a,d]**	**Model 4[a,e]**
**n=9020**		**Coeff (95% CI)**	**Coeff (95% CI)**	**Coeff (95% CI)**	**Coeff (95% CI)**
**Baseline UPF Quintile[f]**	Q1 (Lowest intake)	0 [reference]	0 [reference]	0 [reference]	0 [reference]
	Q2	0.14 (-0.05 to 0.31)	0.14 (-0.05 to 0.31)	0.10 (-0.07 to 0.27)	0.06 (-0.10 to 0.23)
	Q3	0.10 (-0.07 to 0.27)	0.12 (-0.05 to 0.29)	0.03 (-0.13 to 0.20)	0.01 (-0.16 to 0.17)
	Q4	0.14 (-0.04 to 0.31)	0.15 (-0.02 to 0.32)	0.04 (-0.13 to 0.21)	0.02 (-0.15 to 0.19)
	Q5 (Highest intake)	0.29 (0.11 to 0.46)[j]	0.26 (0.08 to 0.43)[j]	0.09 (-0.07 to 0.26)	0.08 (-0.09 to 0.24)
**Age[g]**	per year increment	0.55 (0.53 to 0.56)[j]	0.55 (0.53 to 0.56)[j]	0.55 (0.53 to 0.56)[j]	0.55 (0.53 to 0.56)[j]
**Interaction[h]**	Q1*Age	0 [reference]	0 [reference]	0 [reference]	0 [reference]
	Q2*Age	0.02 (-0.001 to 0.04)	0.02 (-0.001 to 0.04)	0.02 (-0.001 to 0.04)	0.02 (-0.001 to 0.04)
	Q3*Age	0.03 (0.005 to 0.04)[i]	0.03 (0.005 to 0.04)[i]	0.03 (0.005 to 0.04)[i]	0.03 (0.005 to 0.04)[i]
	Q4*Age	0.04 (0.01 to 0.06)[j]	0.04 (0.01 to 0.06)[j]	0.04 (0.01 to 0.06)[j]	0.04 (0.01 to 0.06)[j]
	Q5*Age	0.06 (0.04 to 0.08)[j]	0.06 (0.04 to 0.08)[j]	0.07 (0.04 to 0.08)[j]	0.06 (0.04 to 0.08)[j]
**Fat Mass Index (kg/m²), n=8078**		**Model 1[a,b]**	**Model 2[a,c]**	**Model 3[a,d]**	**Model 4[a,e]**
**Baseline UPF Quintile[f]**	Q1 (Lowest intake)	0 [reference]	0 [reference]	0 [reference]	0 [reference]
	Q2	0.15 (-0.03 to 0.33)	0.17 (-0.01 to 0.34)	0.10 (-0.07 to 0.27)	0.08 (-0.09 to 0.26)
	Q3	0.21 (0.02 to 0.39)[i]	0.25 (0.06 to 0.42)[j]	0.12 (-0.05 to 0.30)	0.11 (-0.06 to 0.28)
	Q4	0.27 (0.08 to 0.46)[j]	0.32 (0.13 to 0.49)[j]	0.18 (-0.003 to 0.35)	0.17 (-0.01 to 0.34)
	Q5 (Highest intake)	0.54 (0.34 to 0.72)[j]	0.51 (0.33 to 0.69)[j]	0.28 (0.10 to 0.46)[j]	0.27 (0.09 to 0.45)[j]
**Age[g]**	per year increment	0.22 (0.20 to 0.23)[j]	0.22 (0.20 to 0.23)[j]	0.22 (0.20 to 0.23)[j]	0.22 (0.20 to 0.23)[j]
**Interaction[h]**	Q1*Age	0 [reference]	0 [reference]	0 [reference]	0 [reference]
	Q2*Age	0.004 (-0.01 to 0.02)	0.005 (-0.01 to 0.02)	0.005 (-0.01 to 0.02)	0.005 (-0.01 to 0.02)
	Q3*Age	0.01 (-0.01 to 0.03)	0.01 (-0.01 to 0.03)	0.01 (-0.01 to 0.02)	0.01 (-0.01 to 0.02)
	Q4*Age	0.01 (-0.01 to 0.03)	0.01 (-0.01 to 0.03)	0.01 (-0.01 to 0.03)	0.01 (-0.01 to 0.03)
	Q5*Age	0.03 (0.01 to 0.05)[j]	0.03 (0.01 to 0.05)[j]	0.03 (0.01 to 0.05)[j]	0.03 (0.01 to 0.05)[j]

Abbreviations: Coeff, coefficient; CI, confidence interval; UPF, ultra-processed food consumption was defined as the proportion of its weight contribution relative to daily food intake measured in g/day and was categorized into quintiles (Q1-Q5 represents lowest to highest quintile of UPF consumption).

[a] Linear growth curve models were employed with individual-specific random intercept and random slope using age as the underlying timescale, and included baseline UPF quintile, an interaction term between age and baseline UPF quintile. Baseline refers to 7 years old for body mass index and 9 years old for fat mass index outcome.

© 2021 Chang K et al. *JAMA Pediatrics*.

[b] Model 1 was not adjusted for any covariates.
[c] Model 2 = Model 1 + child's sex (male/female), ethnicity (white/non-white), birth weight (<2500g/2500-3999g/≥4000g), physical activity (moderate-to-vigorous physical activity per day≥60 minutes/otherwise), quintiles of Index of Multiple Deprivation.
[d] Model 3 = Model 2 + mother's pre-pregnancy BMI (<18.5/18.5-24.9/25-29.9/≥30kg/m^2), marital status (single/married or living with partner), highest educational attainment (CSE or none/vocational/O level/A level/Degree or above), socio-economic status based on UK National Statistics Socioeconomic Classification (higher managerial, administrative and professional occupations/intermediate occupations/routine and manual occupation).
[e] Model 4 = Model 3 + total energy intake (continuous, kcal/day) at baseline.
[f] Coefficient of Baseline UPF quintile: assesses the difference in mean adiposity outcomes at baseline among those of higher UPF consumption quintile compared with the lowest UPF quintile reference group.
[g] Coefficient of Age: captures the average yearly growth in adiposity outcomes for the reference group and were centered at baseline age of each outcome (described above).
[h] Coefficient of Interaction term: examines the difference in average growth trajectories of higher UPF consumption quintile compared with the lowest UPF quintile reference group.
[i] $P < .05$
[j] $P < .01$

© 2021 Chang K et al. *JAMA Pediatrics*.

eTable 4. Stepwise Adjustments for Lean Mass Index and Body Fat Percentage

Lean Mass Index (kg/m^2)		Model 1[a,b]	Model 2[a,c]	Model 3[a,d]	Model 4[a,e]
n=8078		Coeff (95% CI)	Coeff (95% CI)	Coeff (95% CI)	Coeff (95% CI)
Baseline UPF Quintile[f]	Q1 (Lowest intake)	0 [reference]	0 [reference]	0 [reference]	0 [reference]
	Q2	0.04 (-0.03 to 0.10)	0.03 (-0.04 to 0.09)	0.02 (-0.05 to 0.09)	0.005 (-0.06 to 0.07)
	Q3	0.05 (-0.02 to 0.11)	0.04 (-0.03 to 0.10)	0.02 (-0.05 to 0.08)	0.009 (-0.06 to 0.07)
	Q4	0.05 (-0.02 to 0.12)	0.02 (-0.05 to 0.08)	-0.002 (-0.07 to 0.07)	-0.01 (-0.08 to 0.05)
	Q5 (Highest intake)	0.07 (-0.005 to 0.13)	0.03 (-0.04 to 0.09)	-0.004 (-0.07 to 0.06)	-0.01 (-0.08 to 0.05)
Age[g]	per year increment	0.55 (0.53 to 0.55)[j]	0.55 (0.53 to 0.55)[j]	0.55 (0.53 to 0.55)[j]	0.55 (0.53 to 0.55)[j]
Age^2[g]	per year increment	-0.02 (-0.02 to -0.01)[j]	-0.02 (-0.02 to -0.01)[j]	-0.02 (-0.02 to -0.01)[j]	-0.02 (-0.02 to -0.01)[j]
Interaction[h]	Q1*Age	0 [reference]	0 [reference]	0 [reference]	0 [reference]
	Q2*Age	0.008 (-0.003 to 0.01)	0.008 (-0.003 to 0.01)	0.008 (-0.003 to 0.01)	0.008 (-0.003 to 0.01)
	Q3*Age	-0.002 (-0.01 to 0.009)	-0.002 (-0.01 to 0.009)	-0.002 (-0.01 to 0.009)	-0.003 (-0.01 to 0.008)
	Q4*Age	0.009 (-0.003 to 0.02)	0.009 (-0.002 to 0.02)	0.009 (-0.002 to 0.02)	0.009 (-0.002 to 0.02)
	Q5*Age	0.005 (-0.007 to 0.01)	0.004 (-0.007 to 0.01)	0.005 (-0.007 to 0.01)	0.004 (-0.007 to 0.01)

Total fat percentage (%), n=8078		Model 1[a,b]	Model 2[a,c]	Model 3[a,d]	Model 4[a,e]
Baseline UPF Quintile[f]	Q1 (Lowest intake)	0 [reference]	0 [reference]	0 [reference]	0 [reference]
	Q2	0.75 (0.05 to 1.44)[i]	0.88 (0.21 to 1.55)[i]	0.68 (0.01 to 1.33)[i]	0.65 (-0.01 to 1.30)
	Q3	0.90 (0.21 to 1.58)[i]	1.09 (0.43 to 1.74)[j]	0.69 (0.04 to 1.34)[i]	0.67 (0.02 to 1.32)[i]
	Q4	1.15 (0.45 to 1.85)[j]	1.49 (0.82 to 2.16)[j]	1.04 (0.37 to 1.69)[j]	1.02 (0.35 to 1.67)[j]
	Q5 (Highest intake)	2.18 (1.48 to 2.87)[j]	2.22 (1.54 to 2.88)[j]	1.49 (0.83 to 2.15)[j]	1.47 (0.81 to 2.13)[j]
Age[g]	per year increment	0.41 (0.36 to 0.44)[j]	0.39 (0.35 to 0.43)[j]	0.39 (0.35 to 0.43)[j]	0.39 (0.35 to 0.43)[j]
Interaction[h]	Q1*Age	0 [reference]	0 [reference]	0 [reference]	0 [reference]
	Q2*Age	-0.04 (-0.10 to 0.01)	-0.04 (-0.09 to 0.02)	-0.03 (-0.08 to 0.02)	-0.03 (-0.08 to 0.02)
	Q3*Age	-0.02 (-0.07 to 0.03)	-0.02 (-0.07 to 0.03)	-0.02 (-0.07 to 0.03)	-0.02 (-0.07 to 0.03)
	Q4*Age	-0.05 (-0.10 to 0.008)	-0.04 (-0.09 to 0.01)	-0.04 (-0.10 to 0.01)	-0.04 (-0.10 to 0.01)
	Q5*Age	-0.005 (-0.06 to 0.05)	0.005 (-0.05 to 0.06)	0.004 (-0.05 to 0.06)	0.004 (-0.05 to 0.06)

Abbreviations: Coeff, coefficient; CI, confidence interval; UPF, ultra-processed food consumption was defined as the proportion of its weight contribution relative to daily food intake measured in g/day and was categorized into quintiles (Q1-Q5 represents lowest to highest quintile of UPF consumption).

© 2021 Chang K et al. *JAMA Pediatrics*.

[a] Linear growth curve models were employed with individual-specific random intercept and random slope using age (and quadratic age for lean mass index outcome) as the underlying timescale, and included baseline UPF quintile, an interaction term between age and baseline UPF quintile. Baseline refers to 9 years old for lean mass index and body fat percentage outcomes.
[b] Model 1 was not adjusted for any covariates.
[c] Model 2 = Model 1 + child's sex (male/female), ethnicity (white/non-white), birth weight (<2500g/2500-3999g/≥4000g), physical activity (moderate-to-vigorous physical activity per day≥60 minutes/otherwise), quintiles of Index of Multiple Deprivation.
[d] Model 3 = Model 2 + mother's pre-pregnancy BMI (<18.5/18.5-24.9/25-29.9/≥30kg/m^2), marital status (single/married or living with partner), highest educational attainment (CSE or none/vocational/O level/A level/Degree or above), socio-economic status based on UK National Statistics Socioeconomic Classification (higher managerial, administrative and professional occupations/intermediate occupations/routine and manual occupation).
[e] Model 4 = Model 3 + total energy intake (continuous, kcal/day) at baseline.
[f] Coefficient of Baseline UPF quintile: assesses the difference in mean adiposity outcomes at baseline among those of higher UPF consumption quintile compared with the lowest UPF quintile reference group.
[g] Coefficient of Age and Age^2: captures the average yearly growth in adiposity outcomes for the reference group and were centered at baseline age of each outcome (described above).
[h] Coefficient of Interaction term: examines the difference in average growth trajectories of higher UPF consumption quintile compared with the lowest UPF quintile reference group.
[i] $P < .05$
[j] $P < .01$

© 2021 Chang K et al. *JAMA Pediatrics*.

eTable 5. Results of Sensitivity Analyses for Primary Adiposity Outcomes

		Body Mass Index (kg/m^2)	Fat Mass Index (kg/m^2)	Lean Mass Index (kg/m^2)	Body fat percentage (%)
		**Coeff (95% CI)**	**Coeff (95% CI)**	**Coeff (95% CI)**	**Coeff (95% CI)**
Model S1[a]		*n= 9020*	*n= 8078*	*n= 8078*	*n= 8085*
**Baseline UPF Quintile**[h]	Q1	0 [reference]	0 [reference]	0 [reference]	0 [reference]
	Q2	0.08 (-0.08 to 0.25)	0.10 (-0.08 to 0.27)	0.006 (-0.06 to 0.07)	0.68 (0.01 to 1.33)[m]
	Q3	0.04 (-0.12 to 0.21)	0.14 (-0.04 to 0.31)	0.01 (-0.05 to 0.08)	0.73 (0.07 to 1.38)[m]
	Q4	0.07 (-0.10 to 0.24)	0.20 (0.01 to 0.38)[m]	-0.008 (-0.08 to 0.06)	1.09 (0.41 to 1.75)[n]
	Q5	0.14 (-0.03 to 0.32)	0.32 (0.13 to 0.50)[n]	-0.007 (-0.08 to 0.06)	1.56 (0.88 to 2.23)[n]
**Age**[i]	per year	0.55 (0.53 to 0.56)[n]	0.22 (0.20 to 0.23)[n]	0.55 (0.53 to 0.55)[n]	0.39 (0.35 to 0.43)[n]
**Age^2**[i]	per year	-	-	-0.02 (-0.02 to -0.01)[n]	-
**Inter-action**[j]	Q1*Age	0 [reference]	0 [reference]	0 [reference]	0 [reference]
	Q2*Age	0.02 (-0.001 to 0.04)	0.005 (-0.01 to 0.02)	0.008 (-0.003 to 0.01)	-0.03 (-0.08 to 0.02)
	Q3*Age	0.03 (0.005 to 0.04)[m]	0.009 (-0.01 to 0.02)	-0.003 (-0.01 to 0.008)	-0.02 (-0.06 to 0.03)
	Q4*Age	0.04 (0.01 to 0.06)[n]	0.01 (-0.01 to 0.03)	0.009 (-0.002 to 0.02)	-0.04 (-0.10 to 0.01)
	Q5*Age	0.06 (0.04 to 0.08)[n]	0.03 (0.01 to 0.05)[n]	0.004 (-0.007 to 0.01)	0.004 (-0.05 to 0.06)
Model S2[b]		*n= 9020*	*n= 8077*	*n= 8077*	*n= 8084*
**Baseline UPF Quintile**[h]	Q1	0 [reference]	0 [reference]	0 [reference]	0 [reference]
	Q2	0.02 (-0.15 to 0.18)	0.05 (-0.13 to 0.22)	-0.009 (-0.08 to 0.06)	0.56 (-0.09 to 1.22)
	Q3	-0.06 (-0.22 to 0.11)	0.06 (-0.12 to 0.23)	-0.008 (-0.07 to 0.06)	0.54 (-0.11 to 1.19)
	Q4	-0.07 (-0.25 to 0.10)	0.09 (-0.09 to 0.27)	-0.03 (-0.10 to 0.03)	0.84 (0.16 to 1.51)[m]
	Q5	-0.04 (-0.21 to 0.14)	0.17 (-0.02 to 0.35)	-0.03 (-0.10 to 0.04)	1.19 (0.40 to 1.87)[n]
**Age**[i]	per year	0.55 (0.53 to 0.56)[n]	0.22 (0.20 to 0.23)[n]	0.54 (0.53 to 0.55)[n]	0.40 (0.35 to 0.43)[n]
**Age^2**[i]	per year	-	-	-0.02 (-0.02 to -0.01)[n]	-
**Inter-action**[j]	Q1*Age	0 [reference]	0 [reference]	0 [reference]	0 [reference]
	Q2*Age	0.02 (-0.0008 to 0.04)	0.005 (-0.01 to 0.02)	0.008 (-0.003 to 0.01)	-0.03 (-0.08 to 0.02)
	Q3*Age	0.03 (0.005 to 0.04)[m]	0.009 (-0.01 to 0.02)	-0.002 (-0.01 to 0.009)	-0.02 (-0.06 to 0.03)
	Q4*Age	0.04 (0.02 to 0.06)[n]	0.01 (-0.009 to 0.03)	0.01 (-0.002 to 0.02)	-0.04 (-0.10 to 0.01)
	Q5*Age	0.07 (0.04 to 0.08)[n]	0.03 (0.01 to 0.05)[n]	0.005 (-0.007 to 0.01)	0.002 (-0.05 to 0.05)
Model S3[c]		*n= 8326*	*n= 7070*	*n= 7070*	*n= 7076*
**Baseline UPF Quintile**[h]	Q1	0 [reference]	0 [reference]	0 [reference]	0 [reference]
	Q2	0.05 (-0.12 to 0.23)	0.05 (-0.13 to 0.23)	-0.002 (-0.07 to 0.07)	0.53 (-0.16 to 1.21)
	Q3	0.02 (-0.15 to 0.19)	0.09 (-0.09 to 0.27)	-0.001 (-0.07 to 0.07)	0.65 (-0.02 to 1.33)
	Q4	0.04 (-0.14 to 0.21)	0.15 (-0.03 to 0.34)	-0.007 (-0.08 to 0.06)	1.01 (0.30 to 1.70)[n]
	Q5	0.10 (-0.08 to 0.28)	0.28 (0.08 to 0.46)[n]	-0.02 (-0.09 to 0.05)	1.53 (0.83 to 2.22)[n]
**Age**[i]	per year	0.55 (0.53 to 0.56)[n]	0.22 (0.20 to 0.23)[n]	0.55 (0.53 to 0.55)[n]	0.39 (0.35 to 0.43)[n]

© 2021 Chang K et al. *JAMA Pediatrics*.

**Age[2i]**	per year	-	-	-0.02 (-0.02 to -0.01)[n]	-
**Inter-action[j]**	Q1*Age	0 [reference]	0 [reference]	0 [reference]	0 [reference]
	Q2*Age	0.02 (-0.0009 to 0.04)	0.005 (-0.01 to 0.02)	0.007 (-0.004 to 0.01)	-0.03 (-0.08 to 0.02)
	Q3*Age	0.03 (0.004 to 0.04)[m]	0.01 (-0.009 to 0.03)	-0.003 (-0.01 to 0.008)	-0.01 (-0.06 to 0.04)
	Q4*Age	0.04 (0.01 to 0.06)[n]	0.01 (-0.01 to 0.03)	0.009 (-0.003 to 0.02)	-0.04 (-0.09 to 0.01)
	Q5*Age	0.07 (0.04 to 0.08)[n]	0.04 (0.01 to 0.05)[n]	0.003 (-0.009 to 0.01)	0.01 (-0.05 to 0.06)
***Model S4*[d]**		*n= 8777*	*n= 7860*	*n= 7860*	*n= 7865*
**Baseline UPF Quintile[h]**	Q1	0 [reference]	0 [reference]	0 [reference]	0 [reference]
	Q2	0.07 (-0.10 to 0.24)	0.09 (-0.08 to 0.27)	0.007 (-0.06 to 0.07)	0.69 (0.02 to 1.35)[m]
	Q3	0.005 (-0.16 to 0.17)	0.11 (-0.02 to 0.34)	0.005 (-0.06 to 0.07)	0.70 (0.04 to 1.35)[m]
	Q4	0.004 (-0.17 to 0.18)	0.16 (0.07 to 0.43)	-0.02 (-0.09 to 0.05)	1.01 (0.34 to 1.68)[n]
	Q5	0.06 (-0.12 to 0.23)	0.25 (0.08 to 0.46)[n]	-0.01 (-0.08 to 0.06)	1.39 (0.72 to 2.06)[n]
**Age[i]**	per year	0.55 (0.53 to 0.56)[n]	0.22 (0.20 to 0.23)[n]	0.54 (0.53 to 0.55)[n]	0.39 (0.35 to 0.43)[n]
**Age[2i]**	per year	-	-	-0.02 (-0.02 to -0.01)[n]	-
**Inter-action[j]**	Q1*Age	0 [reference]	0 [reference]	0 [reference]	0 [reference]
	Q2*Age	0.02 (0.001 to 0.04)[m]	0.006 (-0.01 to 0.02)	0.01 (-0.001 to 0.02)	-0.03 (-0.08 to 0.02)
	Q3*Age	0.03 (0.005 to 0.04)[m]	0.009 (-0.01 to 0.02)	-0.0007 (-0.01 to 0.01)	-0.02 (-0.07 to 0.03)
	Q4*Age	0.04 (0.02 to 0.06)[n]	0.01 (-0.01 to 0.03)	0.01 (-0.004 to 0.02)	-0.04 (-0.10 to 0.01)
	Q5*Age	0.07 (0.04 to 0.09)[n]	0.04 (0.01 to 0.05)[n]	0.005 (-0.006 to 0.01)	0.01 (-0.04 to 0.06)
***Model S5*[e]**		*n= 4541*	*n= 3976*	*n= 3976*	*n= 3980*
**Baseline UPF Quintile[h]**	Q1	0 [reference]	0 [reference]	0 [reference]	0 [reference]
	Q2	0.13 (-0.10 to 0.36)	0.13 (-0.12 to 0.38)	0.05 (-0.06 to 0.15)	0.67 (-0.30 to 1.64)
	Q3	0.09 (-0.13 to 0.31)	0.14 (-0.10 to 0.39)	0.09 (-0.02 to 0.19)	0.72 (-0.22 to 1.67)
	Q4	-0.09 (-0.31 to 0.13)	0.04 (-0.21 to 0.29)	-0.05 (-0.15 to 0.06)	0.53 (-0.43 to 1.49)
	Q5	0.05 (-0.18 to 0.27)	0.32 (0.06 to 0.58)[n]	-0.05 (-0.16 to 0.05)	1.75 (0.76 to 2.72)[n]
**Age[i]**	per year	0.56 (0.54 to 0.58)[n]	0.12 (0.10 to 0.14)[n]	0.82 (0.80 to 0.83)[n]	0.07 (0.006 to 0.12)[m]
**Age[2i]**	per year	-	-	-0.03 (-0.03 to -0.02)[n]	-
**Inter-action[j]**	Q1*Age	0 [reference]	0 [reference]	0 [reference]	0 [reference]
	Q2*Age	0.02 (-0.01 to 0.04)	-0.002 (-0.02 to 0.02)	-0.0002 (-0.01 to 0.01)	-0.04 (-0.11 to 0.04)
	Q3*Age	0.02 (-0.01 to 0.04)	0.003 (-0.02 to 0.02)	-0.01 (-0.02 to 0.002)	-0.02 (-0.10 to 0.06)
	Q4*Age	0.03 (0.001 to 0.06)[m]	0.01 (-0.01 to 0.03)	0.002 (-0.01 to 0.01)	-0.02 (-0.10 to 0.06)
	Q5*Age	0.05 (0.02 to 0.08)[n]	0.02 (-0.006 to 0.04)	-0.01 (-0.02 to 0.005)	-0.01 (-0.09 to 0.07)
***Model S6*[f]**		*n= 4479*	*n= 4102*	*n= 4102*	*n= 4105*
**Baseline UPF Quintile[h]**	Q1	0 [reference]	0 [reference]	0 [reference]	0 [reference]
	Q2	-0.01 (-0.27 to 0.24)	0.03 (-0.22 to 0.27)	-0.05 (-0.15 to 0.04)	0.46 (-0.38 to 1.30)
	Q3	-0.11 (-0.36 to 0.14)	0.04 (-0.20 to 0.28)	-0.06 (-0.16 to 0.03)	0.35 (-0.48 to 1.18)
	Q4	0.11 (-0.15 to 0.37)	0.24 (-0.01 to 0.49)	0.004 (-0.09 to 0.10)	1.13 (0.26 to 1.99)[m]

© 2021 Chang K et al. *JAMA Pediatrics*.

	Q5	0.10 (-0.16 to 0.36)	0.23 (-0.02 to 0.48)	-0.03 (-0.12 to 0.07)	1.07 (0.20 to 1.92)[m]
**Age**[i]	per year	0.54 (0.52 to 0.56)[n]	0.30 (0.27 to 0.31)[n]	0.31 (0.29 to 0.31)[n]	0.67 (0.62 to 0.71)[n]
**Age^2**[i]	per year	-	-	-0.008 (-0.008 to -0.007)[n]	-
**Inter-action**[j]	Q1*Age	0 [reference]	0 [reference]	0 [reference]	0 [reference]
	Q2*Age	0.03 (-0.005 to 0.05)	0.02 (-0.01 to 0.04)	0.01 (0.001 to 0.02)[m]	-0.02 (-0.08 to 0.04)
	Q3*Age	0.04 (0.004 to 0.06)[m]	0.02 (-0.004 to 0.04)	0.003 (-0.006 to 0.01)	0.02 (-0.04 to 0.08)
	Q4*Age	0.05 (0.01 to 0.08)[n]	0.02 (-0.004 to 0.05)	0.005 (-0.005 to 0.01)	-0.03 (-0.09 to 0.03)
	Q5*Age	0.07 (0.04 to 0.10)[n]	0.04 (0.01 to 0.07)[n]	0.01 (0.004 to 0.02)[m]	0.02 (-0.04 to 0.08)
Model S7[g]		*n= 9020*	*n= 8078*	*n= 8078*	*n= 8085*
**Baseline UPF Quintile**[k]	G1	-0.09 (-0.35 to 0.17)	-0.18 (-0.45 to 0.08)	-0.06 (-0.17 to 0.04)	-0.95 (-1.94 to 0.04)
	G2	0 [reference]	0 [reference]	0 [reference]	0 [reference]
	G3	-0.002 (-0.12 to 0.12)	0.10 (-0.03 to 0.22)	-0.008 (-0.05 to 0.04)	0.49 (0.01 to 0.95)[m]
	G4	0.009 (-0.15 to 0.12)	0.16 (-0.01 to 0.33)	-0.02 (-0.09 to 0.04)	0.88 (0.24 to 1.51)[n]
	G5	-0.17 (-0.57 to 0.23)	0.22 (-0.20 to 0.65)	-0.13 (-0.30 to 0.03)	1.63 (0.06 to 3.20)[m]
**Age**[i]	per year	0.57 (0.55 to 0.57)[n]	0.22 (0.21 to 0.23)[n]	0.55 (0.53 to 0.55)[n]	0.37 (0.34 to 0.40)[n]
**Age^2**[i]	per year	-	-	-0.02 (-0.02 to -0.01)[n]	-
**Inter-action**[l]	Age*G1	-0.04 (-0.07 to -0.01)[n]	-0.006 (-0.03 to 0.02)	-0.01 (-0.03 to 0.003)	0.05 (-0.02 to 0.13)
	Age*G2	0 [reference]	0 [reference]	0 [reference]	0 [reference]
	Age*G3	0.02 (0.005 to 0.03)[n]	0.009 (-0.005 to 0.02)	-0.0003 (-0.008 to 0.008)	-0.005 (-0.04 to 0.03)
	Age*G4	0.05 (0.03 to 0.07)[n]	0.03 (0.01 to 0.05)[n]	0.002 (-0.009 to 0.01)	0.03 (-0.02 to 0.08)
	Age*G5	0.09 (0.03 to 0.14)[n]	0.03 (-0.01 to 0.08)	0.002 (-0.02 to 0.03)	-0.005 (-0.15 to 0.14)

Abbreviations: Coeff, coefficient; CI, confidence interval; UPF, ultra-processed food consumption was defined as the proportion of its weight contribution relative to daily food intake measured in g/day and was categorized into quintiles (Q1-Q5 represents lowest to highest quintile of UPF consumption).
[a] Model S1: Fully adjusted model (Table 2) and additionally include the intake of fruit and vegetable (g/day).
[b] Model S2: Fully adjusted model (Table 2) and additionally include the intake of saturated fat (g/day), sugar (g/day), fiber (g/day) and sodium (mg/day).
[c] Model S3: Fully adjusted model (Table 2) but excluded children with no follow-up data.
[d] Model S4: Fully adjusted model (Table 2) but excluded twin children.
[e] Model S5: Fully adjusted model (Table 2) for boys only.
[f] Model S6: Fully adjusted model (Table 2) for girls only.
[g] Model S7: Re-categorized baseline UPF consumption into 5 groups: G1 with <20%; G2 with 20%-39%; G3 with 40%-59%; G4 with 60%-79%; and G5 with ≥80% of daily food intake (g/day) sourced from ultra-processed foods. Sample sizes of these 5 intake groups were 419, 3278, 3782, 1368 and 178, respectively.
[h] Coefficient of Baseline UPF quintile: assesses the difference in mean adiposity outcomes at baseline among those of higher UPF consumption quintile compared with the lowest UPF quintile reference group (Q1). Baseline refers to 7 years old for body mass index and 9 years old for fat mass index outcomes.
[i] Coefficient of Age and Age^2: captures the average yearly growth in adiposity outcomes for the reference group and were centred at baseline age of each outcome (described above).
[j] Coefficient of Interaction term: examines the difference in average growth trajectories of higher UPF consumption quintile compared with the lowest UPF quintile reference group (Q1*Age).
[k] Coefficient of Baseline UPF%: assesses the difference in mean adiposity outcomes at baseline among those assigned to respective UPF intake group compared with the 20%-40% UPF intake reference group.
[l] Coefficient of Interaction term: examines the difference in average growth trajectories of those assigned to respective UPF intake group compared with the 20%-40% UPF intake reference group.
[m] *P* <.05
[n] *P* <.01

© 2021 Chang K et al. *JAMA Pediatrics*.

eTable 6. Results of Growth-Curve Model Using Secondary Exposure[a]

		Body Mass Index (kg/m^2)	BMI z-score	Fat Mass Index (kg/m^2)	Fat Mass (kg)	Total fat percentage (%)
		n=9020	*n=9018*	*n=8078*	*n=8075*	*n=8085*
		Coeff (95% CI)	Coeff (95% CI)	Coeff (95% CI)	Coeff (95% CI)	Coeff (95% CI)
**Baseline UPF Quintile**[b]	Q1	0 [reference]	0 [reference]	0 [reference]	0 [reference]	0 [reference]
	Q2	0.02 (-0.15, 0.19)	-0.0003 (-0.07, 0.07)	0.09 (-0.09, 0.26)	0.12 (-0.30, 0.55)	0.49 (-0.18, 1.15)
	Q3	-0.07 (-0.23, 0.10)	-0.03 (-0.09, 0.04)	0.02 (-0.15, 0.20)	-0.07 (-0.49, 0.35)	0.17 (-0.48, 0.83)
	Q4	-0.04 (-0.20, 0.13)	0.001 (-0.07, 0.07)	0.06 (-0.11, 0.23)	0.06 (-0.35, 0.47)	0.42 (-0.22, 1.07)
	Q5	-0.07 (-0.24, 0.10)	-0.04 (-0.11, 0.03)	0.08 (-0.10, 0.25)	0.04 (-0.38, 0.46)	0.72 (0.05, 1.37)[e]
**Age**[c]	per year	0.56 (0.54, 0.57)f	0.02 (0.01, 0.02)[f]	0.22 (0.20, 0.23)[f]	0.98 (0.93, 1.02)[f]	0.38 (0.34, 0.41)[f]
**Interaction**[d]	Q1*Age	0 [reference]	0 [reference]	0 [reference]	0 [reference]	0 [reference]
	Q2*Age	-0.005 (-0.02, 0.01)	0.001 (-0.005, 0.008)	-0.007 (-0.02, 0.01)	-0.009 (-0.06, 0.05)	-0.04 (-0.09, 0.01)
	Q3*Age	0.02 (0.001, 0.04)[e]	0.004 (-0.003, 0.01)	0.005 (-0.01, 0.02)	0.05 (-0.01, 0.10)	-0.01 (-0.06, 0.04)
	Q4*Age	0.03 (0.006, 0.05)[e]	0.006 (-0.001, 0.01)	0.01 (-0.007, 0.03)	0.06 (-0.0001, 0.12)	-0.002 (-0.05, 0.05)
	Q5*Age	0.05 (0.02, 0.07)[f]	0.01 (0.005, 0.01)[f]	0.04 (0.01, 0.05)[f]	0.14 (0.07, 0.19)[f]	0.04 (-0.01, 0.09)
		**Weight (kg)**	**Weight (kg)**	**Lean Mass Index (kg/m^2)**	**Lean Mass (kg)**	
		n=9021	*n=9021*	*n=8078*	*n=8075*	
**Baseline UPF Quintile**[b]	Q1	0 [reference]	0 [reference]	0 [reference]	0 [reference]	
	Q2	-0.09 (-0.43, 0.25)	-0.19 (-0.59, 0.22)	-0.05 (-0.12, 0.02)	-0.08 (-0.37, 0.22)	
	Q3	-0.17 (-0.51, 0.16)	-0.29 (-0.69, 0.11)	-0.03 (-0.09, 0.04)	-0.07 (-0.36, 0.21)	
	Q4	0.11 (-0.22, 0.45)	-0.31 (-0.71, 0.08)	0.01 (-0.06, 0.07)	0.04 (-0.25, 0.33)	
	Q5	-0.18 (-0.52, 0.16)	-0.22 (-0.63, 0.18)	-0.04 (-0.11, 0.03)	-0.18 (-0.47, 0.11)	
**Age**[c]	per year	5.49 (5.41, 5.55)[f]	3.39 (3.33, 3.43)f	0.55 (0.53, 0.56)[f]	4.47 (4.40, 4.52)[f]	
**Age^2**[c]	per year	-0.12 (-0.12, -0.11)f	-0.11 (-0.11, -0.10)f	-0.02 (-0.02, -0.01)f	-0.17 (-0.17, -0.16)f	
**Interaction**[d]	Q1*Age	0 [reference]	0 [reference]	0 [reference]	0 [reference]	
	Q2*Age	0.005 (-0.07, 0.08)	0.007 (-0.05, 0.06)	-0.003 (-0.01, 0.009)	-0.03 (-0.10, 0.03)	
	Q3*Age	0.07 (-0.01, 0.15)	0.04 (-0.01, 0.09)	0.0004 (-0.01, 0.01)	0.008 (-0.05, 0.07)	
	Q4*Age	0.05 (-0.03, 0.13)	0.09 (0.03, 0.14)[f]	-0.003 (-0.01, 0.009)	-0.05 (-0.11, 0.02)	
	Q5*Age	0.14 (0.06, 0.22)[f]	0.11 (0.05, 0.16)[f]	-0.002 (-0.01, 0.009)	-0.05 (-0.12, 0.01)	

Abbreviations: Coeff, coefficient; CI, confidence interval; UPF, ultra-processed food consumption was computed as the proportion of its calorie contribution relative to total energy intake (kcal/day) and was categorized into quintiles (Q1-Q5 represents lowest to highest quintile of UPF consumption).

© 2021 Chang K et al. *JAMA Pediatrics*.

[a] Linear growth curve models were employed with individual-specific random intercept and random slope using age (and quadratic age where appropriate) as the underlying timescale, and included baseline UPF quintile, an interaction term between age and baseline UPF quintile, and were further adjusted by child's sex (male/female), ethnicity (white/non-white), birth weight (<2500g/2500-3999g/≥4000g), physical activity (moderate-to-vigorous physical activity per day≥60 minutes/otherwise), quintiles of Index of Multiple Deprivation, mother's pre-pregnancy BMI (<18.5/18.5-24.9/25-29.9/≥30kg/m^2), marital status (single/married or living with partner), highest educational attainment (CSE or none/vocational/O level/A level/Degree or above), socio-economic status based on UK National Statistics Socioeconomic Classification (higher managerial, administrative and professional/intermediate/routine and manual occupation), and child's total energy intake (continuous, kcal/day) at baseline. Baseline refers to 7 years old for body mass index (BMI), BMI z-score, weight and waist circumference outcomes; and 9 years old for fat/lean mass index, fat/lean mass and body fat percentage outcomes.
[b] Coefficient of Baseline UPF quintile: assesses the difference in mean adiposity outcomes at baseline among those of higher UPF consumption quintile compared with the lowest UPF quintile reference group.
[c] Coefficient of Age and Age^2: captures the average yearly growth in adiposity outcomes for the reference group and were centered at baseline age of each outcome (described above).
[d] Coefficient of Interaction term: examines the difference in average growth trajectories of higher UPF consumption quintile compared with the lowest UPF quintile reference group.
[e] $P < .05$
[f] $P < .01$

© 2021 Chang K et al. *JAMA Pediatrics*.

eFigure 1. Sources of Ultraprocessed Foods and Minimally Processed Foods

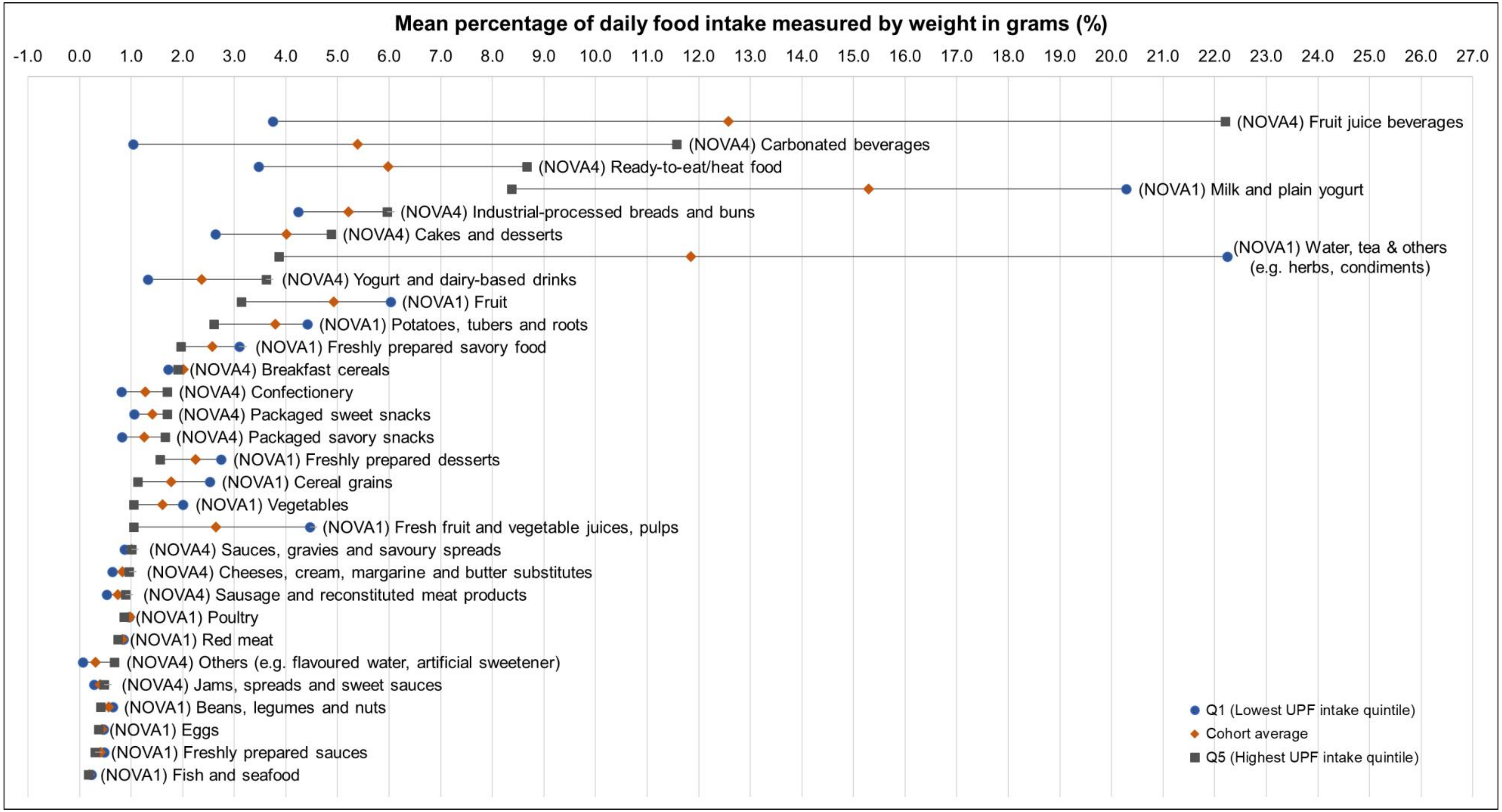

Abbreviation: UPF, ultra-processed food consumption was defined as the proportion of its weight contribution relative to daily food intake measured in g/day and was categorized into quintiles (Q1-Q5 represents lowest to highest quintile of UPF consumption).

© 2021 Chang K et al. *JAMA Pediatrics*.

eFigure 2. Flowchart for Study Cohort Inclusion

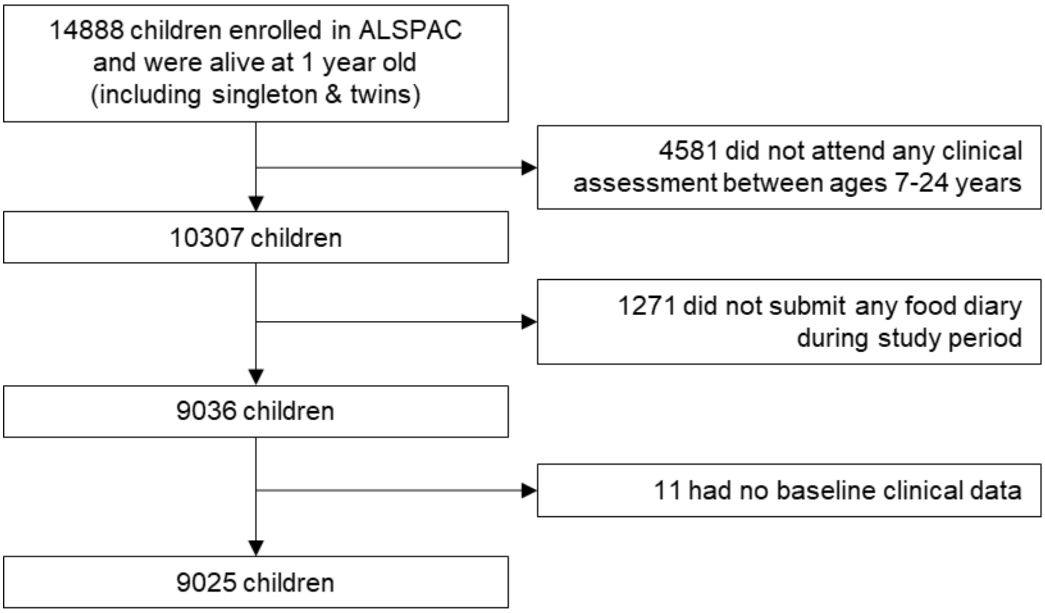

© 2021 Chang K et al. *JAMA Pediatrics*.

eFigure 3. Mean Percentage of Daily Food Intake Stratified by Quintiles of Ultraprocessed Food Consumption

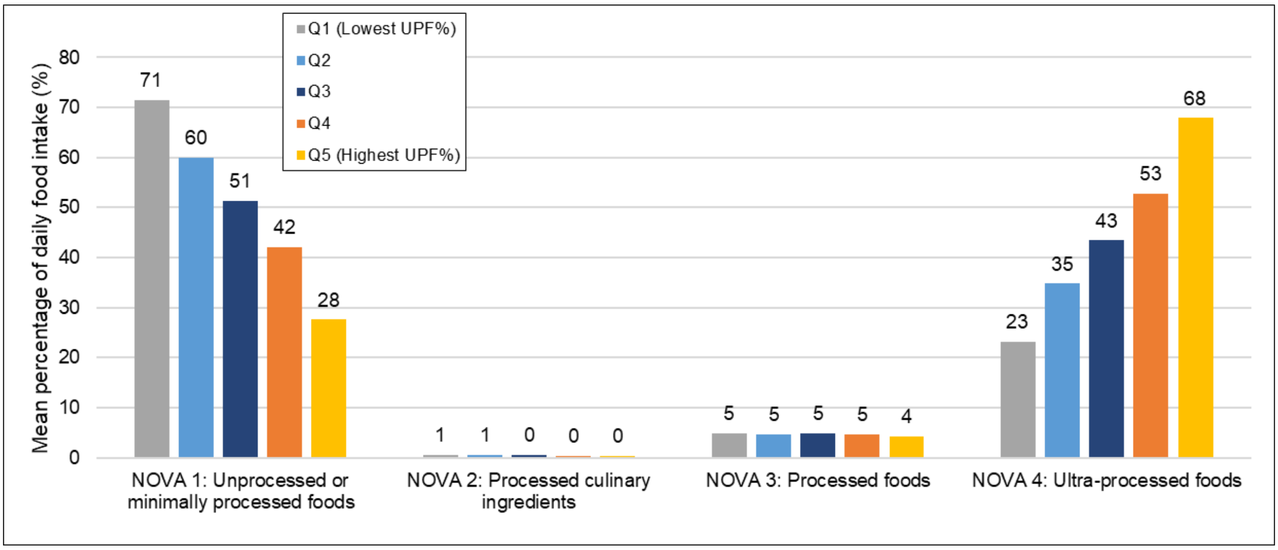

Abbreviation: UPF%, percentage of daily food intake (g/d) contributed by ultra-processed foods (UPFs) at baseline and was categorized into quintiles (Q1-Q5 represents lowest to highest quintile of UPF consumption).

© 2021 Chang K et al. *JAMA Pediatrics*.

eFigure 4. Trajectories of Body Mass Index Stratified by Sex

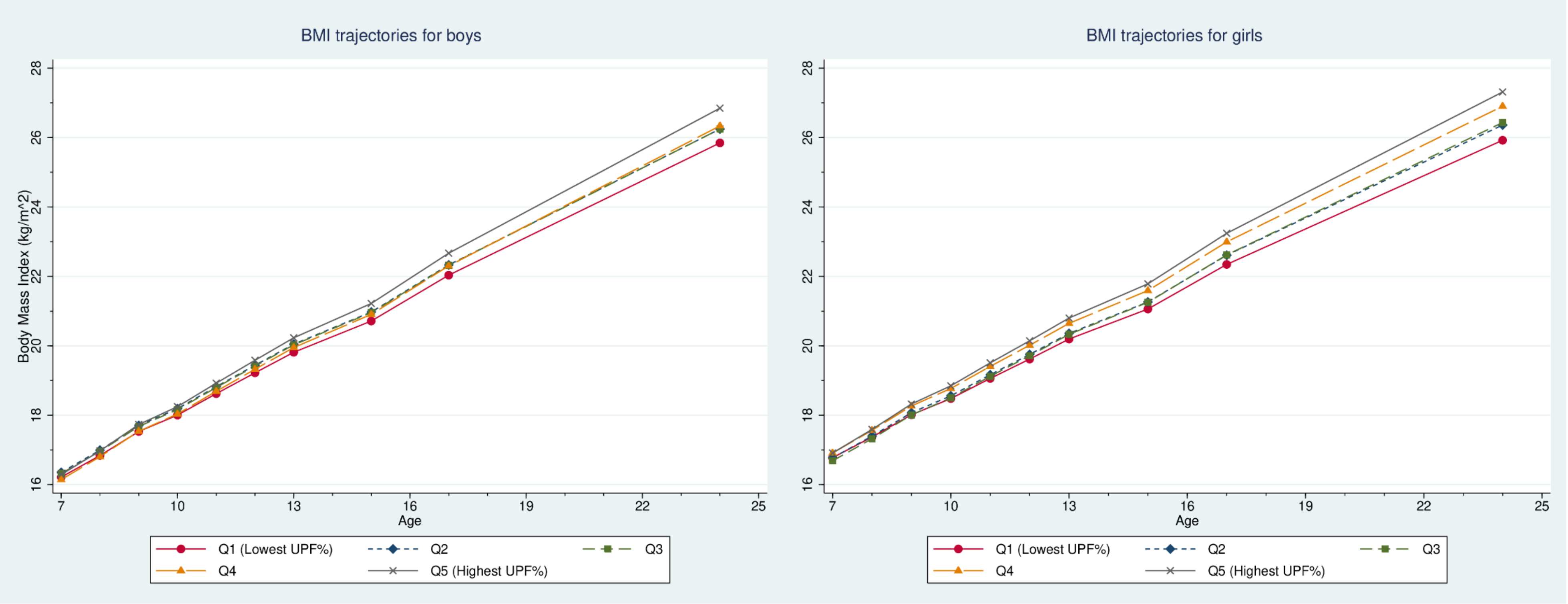

Abbreviations: UPF%, percentage of daily food intake (g/day) contributed by ultra-processed foods (UPFs) at baseline and was further categorized into quintiles (Q1-Q5 represents lowest to highest quintile of UPF consumption). Trajectories were plotted for the predicted values estimated from the growth curve models at each age (wave) of clinic assessment. All linear growth models were fitted with individual-specific random intercept and random slope using age as the underlying timescale, and included baseline UPF quintile, an interaction term between age and baseline UPF quintile, and were further adjusted by ethnicity (white/non-white), birth weight (<2500g/2500-3999g/≥4000g), physical activity (moderate-to-vigorous physical activity per day≥60 minutes/otherwise), quintiles of Index of Multiple Deprivation, mother's pre-pregnancy BMI (<18.5/18.5-24.9/25-29.9/≥30kg/m^2), marital status (single/married or living with partner), highest educational attainment (CSE or none/vocational/O level/A level/Degree or above), socio-economic status based on UK National Statistics Socioeconomic Classification (higher managerial, administrative and professional/intermediate/routine and manual occupation), and child's total energy intake (continuous, kcal/day) at baseline**.** Baseline refers to 7 years old for body mass index outcome.

© 2021 Chang K et al. *JAMA Pediatrics*.

eFigure 5. Sources of Ultraprocessed Foods and Minimally Processed Foods Based on Dietary Energy Intake

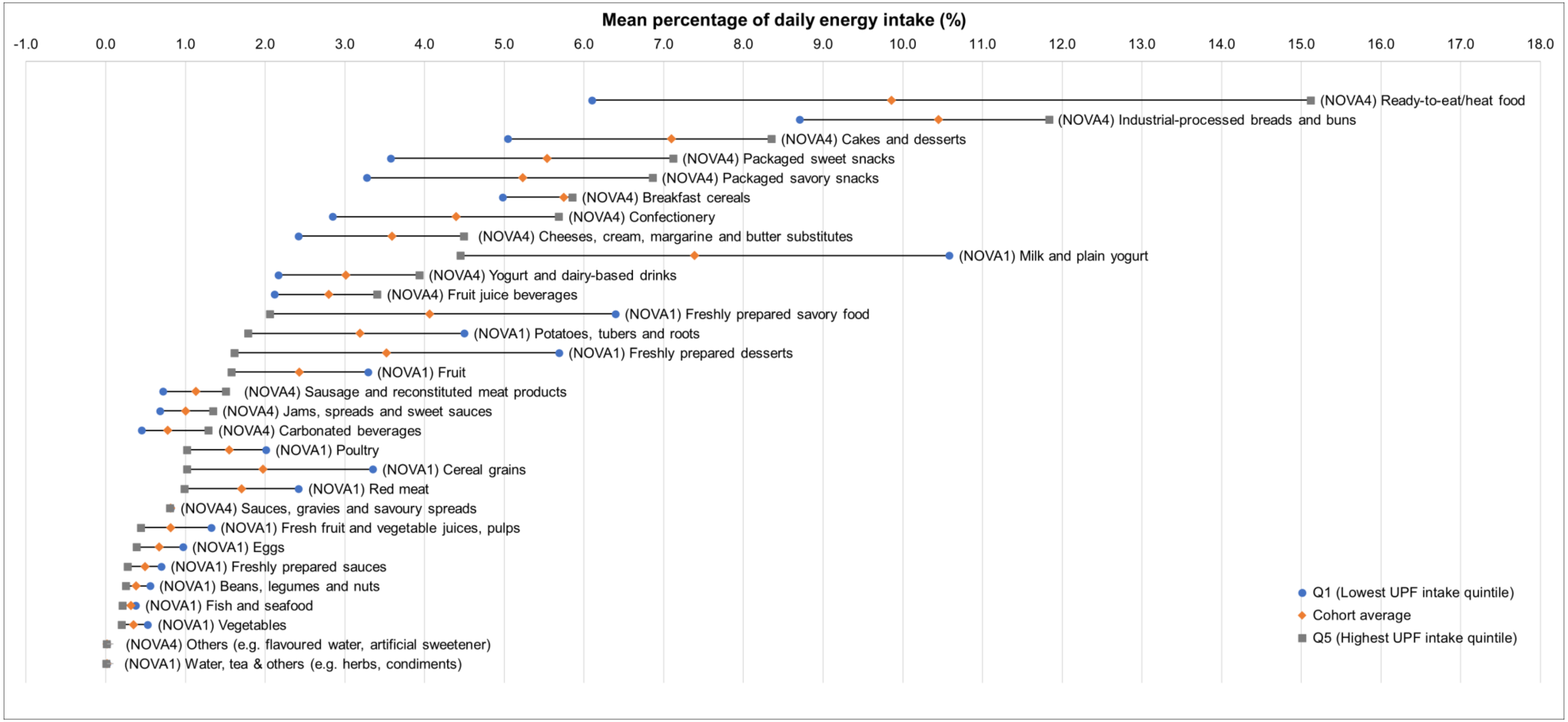

Abbreviation: UPF, ultra-processed food consumption was computed as the proportion of its calorie contribution relative to daily energy intake and was categorized into quintiles (Q1-Q5 represents lowest to highest quintile of UPF consumption).

© 2021 Chang K et al. *JAMA Pediatrics*.

eFigure 6. Results of Growth-Curve Model Using Secondary Exposure

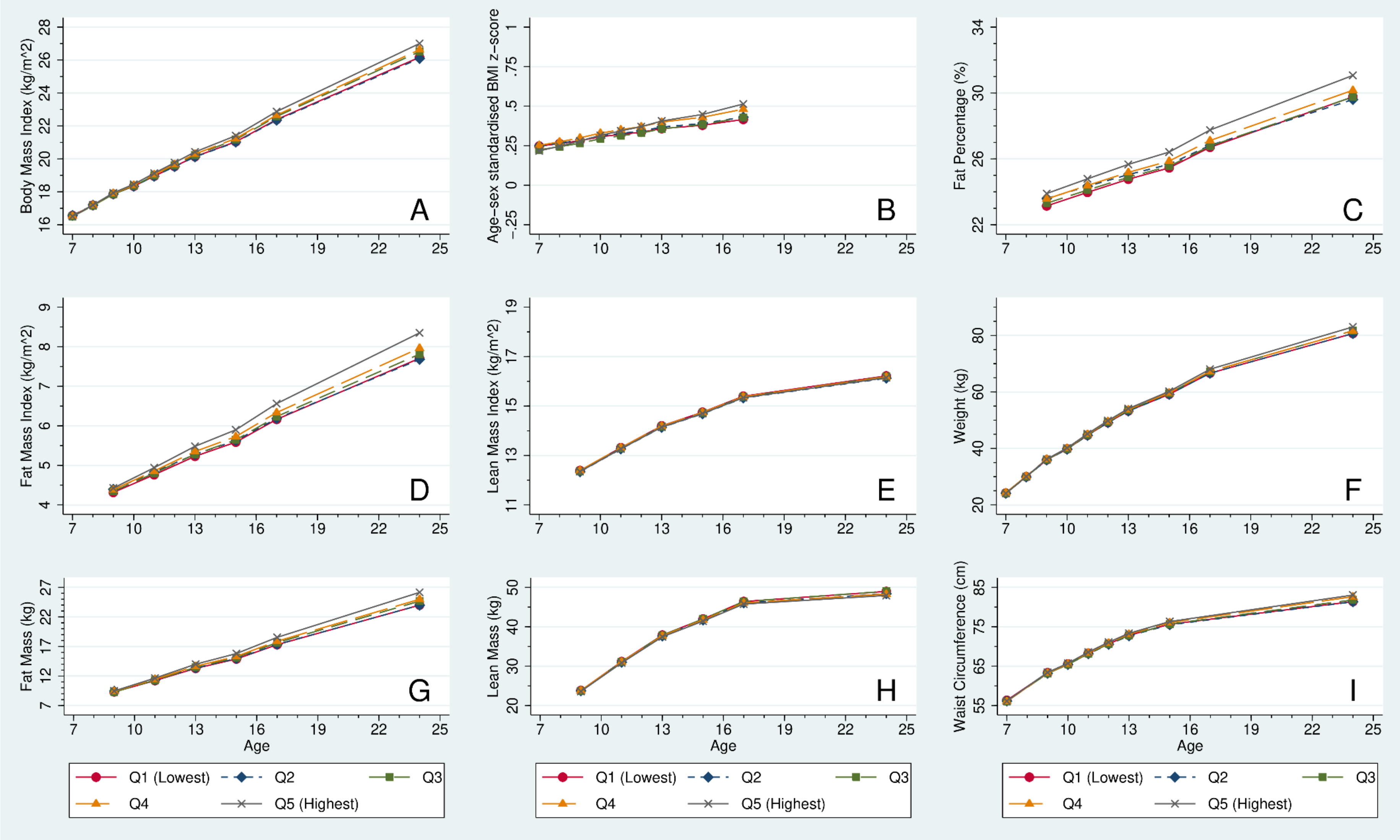

Abbreviation: UPF, ultra-processed food consumption was computed as the proportion of its calorie contribution relative to total energy intake (kcal/day) and was categorized into quintiles (Q1-Q5 represents lowest to highest UPF intake). Figures are labelled as the following: (A) body mass index (BMI); (B) Age-sex standardized BMI z-score; (C) Total fat percentage; (D) Fat mass index; (E) Lean mass index; (F) weight; (G) Fat mass; (H) Lean mass and (I) waist circumference. Linear growth curve models were employed with individual-specific random intercept and random slope using age (and quadratic age where appropriate) as the underlying timescale, and included baseline UPF quintile, an interaction term between age and baseline UPF quintile, and were further adjusted by child's sex (male/female), ethnicity (white/non-white), birth weight (<2500g/2500-3999g/≥4000g), physical activity (moderate-to-vigorous physical activity per day≥60 minutes/otherwise), quintiles of Index of Multiple Deprivation, mother's pre-pregnancy BMI (<18.5/18.5-24.9/25-29.9/≥30kg/m^2), marital status (single/married or living with partner), highest educational attainment (CSE or none/vocational/O level/A level/Degree or above), socio-economic status based on UK National Statistics Socioeconomic Classification (higher managerial,

© 2021 Chang K et al. *JAMA Pediatrics*.

administrative and professional/intermediate/routine and manual occupation), and child's total energy intake (continuous, kcal/day) at baseline**.** Baseline refers to 7 years old for body mass index (BMI), BMI z-score, weight and waist circumference outcomes; and 9 years old for fat/lean mass index, fat/lean mass and body fat percentage outcomes.

© 2021 Chang K et al. *JAMA Pediatrics*.
